# Androgen suppresses testicular cancer cell growth *in vitro* and *in vivo*

**DOI:** 10.18632/oncotarget.9109

**Published:** 2016-04-29

**Authors:** Hideo Nakagawa, Takashi Ueda, Saya Ito, Takumi Shiraishi, Hidefumi Taniguchi, Naruhiro Kayukawa, Hiroyuki Nakanishi, So Ushijima, Motohiro Kanazawa, Terukazu Nakamura, Yoshio Naya, Fumiya Hongo, Kazumi Kamoi, Koji Okihara, Osamu Ukimura

**Affiliations:** ^1^ Department of Urology, Graduate School of Medical Science, Kyoto Prefectural University of Medicine, Kyoto 602-8566, Japan

**Keywords:** androgen, androgen receptor, testicular cancer, seminoma, tryptophan hydroxylase 1

## Abstract

Silencing of androgen receptor (AR)-meditated androgen signaling is thought to be associated with the development of testicular germ cell tumors (TGCTs). However, the role of the androgen/AR signal in TGCT development has not been investigated. In this study, we show that the androgen/AR signal suppressed the cell growth of seminomas (SEs), a type of TGCT, *in vitro* and *in vivo*. Growth of SE cells was suppressed by DHT treatment and reduction of androgen levels by surgical castration promoted cancer cell growth in an *in vivo* xenograft model. Tryptophan hydroxylase 1 (TPH1), the rate limit enzyme in serotonin synthesis, was one of the genes which expression was reduced in DHT-treated SE cells. TPH1 was highly expressed in SE cancer tissues compared with adjacent normal tissues. Activation of androgen/AR signaling in SE cells reduced the expression of TPH1 in SE cells, followed by the reduction of serotonin secretion in cell culture supernatant. These results suggested that silencing of androgen/AR signaling may cause initiation and progression of SE through increase in *TPH1* gene expression level.

## INTRODUCTION

Androgens exert a wide variety of biological effects in many tissues through the androgen receptor (AR) [1–3]. The AR is a member of the nuclear steroid hormone receptor superfamily and acts as a ligand-dependent transcription factor [4]. Androgen/AR signaling has a critical role in the development and maintenance of the male reproductive organs [5] and in pathological events [6]. For example, moderate activation of androgen/AR signaling is essential for normal prostate development [7]. Additionally, initiation and progression of prostate cancers appear to be coupled with aberrant activation of AR signaling [8]. Activation of androgen/AR signaling has a positive effect on prostate cancer cell growth *in vitro* and *in vivo* [9, 10]. Moreover, these reports suggest that tumor development in other male reproductive organs is dependent on androgen signaling. In testes, moderate androgen/AR signaling is also known to be indispensable for normal development and function [5]. However, the role of androgen/AR signaling in testicular germ-cell tumors (TGCTs) remains unclear.

TGCTs are the most common cancers in young men and can be histologically divided into two groups, seminomas (SEs) and non-seminomas (NSEs). NSEs include many cell types, such as embryonal carcinomas, teratomas, yolk sac carcinomas, and choriocarcinomas [11]. In SEs, there are several epidemiological observations that suggest the association of the incidence of SEs with the androgen/AR signal. In fact, the incidence of SE in Africans, in which androgen levels in the blood are higher than in Caucasians, is significantly lower than that in Caucasians [12]. Furthermore, the risk of SE is high in patients with androgen-insensitivity syndrome (AIS), a condition associated with aberrant repression of the AR signal due to loss-of-function mutations in the *AR* gene [13]. These evidences suggest the possibility that androgen/AR signaling is associated with the development of SE.

In this study, we investigated the effects of androgen/AR signaling on testicular cancer cell growth *in vitro* and *in vivo*.

## RESULTS

### AR was highly expressed in SE cells than in NSE cells

**Figure 1:**
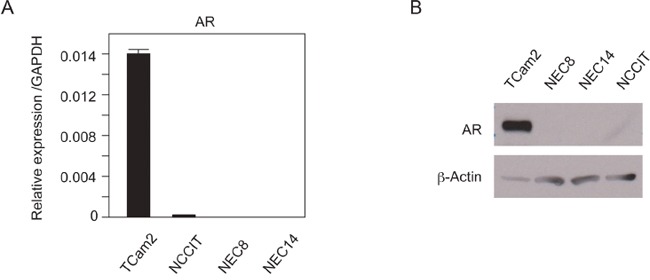
AR expression in TGCT cell lines **A.** mRNA expression levels of AR in four types of TGCT cells were examined by real-time quantitative RT-PCR. The expression of AR was normalized to the GAPDH. Data are presented as mean ± s.d. (n=2). **B.** AR protein levels in TGCT cell lines. Western blots were performed using whole cell lysates extracted from each cell type. The same results were reproduced for each experiment three times.

We used testicular cancer cell lines as a human testicular cancer model to investigate the role of androgen/AR signaling in human testicular cancer. TCam-2 cells were derived from SE cells [14]. NEC8, NEC14 [15], and NCCIT [16] cells were derived from NSE cells. *AR* mRNA expression levels in the cell lines were quantified by reverse transcription polymerase chain reaction (RT-PCR; Figure 1A). The expression levels of *AR* mRNA were significantly higher in TCam-2 cells than in NSE cell lines. AR protein levels were also significantly higher in TCam-2 cells than in NSE cells (Figure 1B).

### Activation of androgen/AR signal suppressed cell growth of SE cells

**Figure 2:**
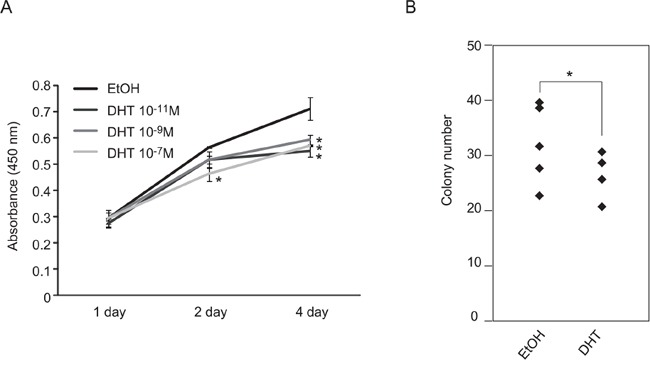
Suppression of TCam-2 cell proliferation by DHT treatment **A.** TCam-2 cell proliferation was observed with or without DHT. 10,000 cells of TCam-2 were plated and cultured with DHT or without DHT (DHT: 10^−7^, 10^−9^ or 10^−11^ M) for four days. The number of living cells was examined by measurement of absorbance (450 nm) at 1, 2 and 4 days after cell seeding. Data are presented as mean ± s.d. (n=3). **B.** Colony formation assays using TCam-2 cells. Cells were cultured with or without DHT (DHT: 10^−7^ M) for 2 weeks. Colony numbers in each dish (n = 5) were visually counted and presented using a column graph. Each experiment was performed three times.* *p* < 0.01.

The gene expression signature of *AR* in the testicular cancer cells may suggest that androgen/AR functions in SE cells. Therefore, the effects of androgen/AR signal activation on TGCT cell growth were examined using SE and NSE cells. Activation of androgen/AR signal following the addition of androgen suppressed cell growth of TCam-2 cells (Figure 2A and 2B). The suppressive effects of the androgen/AR signal were not observed in AR-negative NSE cell lines (Supplemental Figure 1A). These results suggested that androgen/AR signal suppressed SE cell growth *in vitro*.

### Suppression of androgen/AR signal promoted SE cell growth in mice

**Figure 3:**
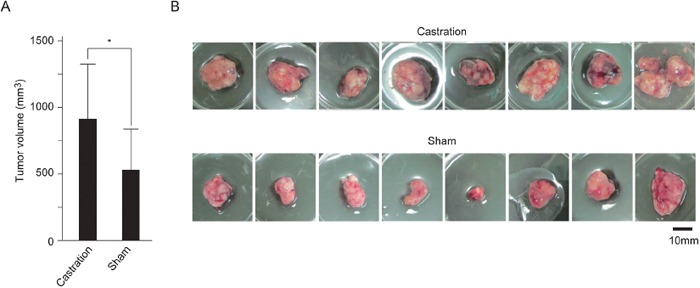
Promotion of SE cell growth in castrated mice **A.** and **B.** Castration or sham operation was performed in 5-week-old male SCID mice. TCam-2 cells were subcutaneously implanted on the right dorsal gluteal region of these mice. After 45 days, the tumors were resected (B). The average volumes of resected tumors in castrated mice (n = 8) and sham-operated mice (n = 8) are shown (A). * *p* < 0.05.

Next, we examined the effect of androgen/AR signal on SE cell growth *in vivo* using mouse xenograft model. TCam-2 cells were implanted under the back skin of SCID mice. On the same day, castration or sham operation was performed. Tumor sizes were evaluated after 45 days. Tumor sizes in castrated mice were larger than those in sham-operated mice (Figure 3A and 3B). These results suggested that suppression of androgen/AR signal increased SE cell growth *in vivo*.

### TPH1 was highly expressed in SE patients and decreased by DHT treatment in SE cells

**Figure 4:**
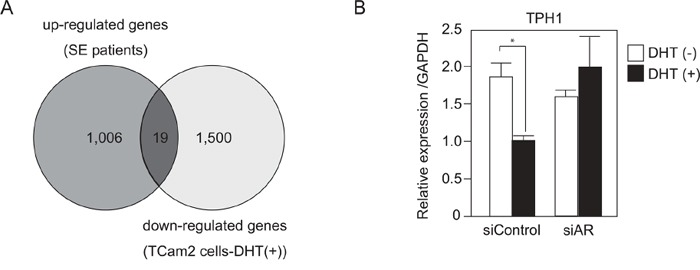
TPH1 was highly expressed in SE patients and down-regulated by DHT in SE cells **A.** Using human SE tumor samples (Table S1), we examined the gene expression profile of these cells by Agilent Microarray technology. 1,006 genes (dark gray circle) exhibited a more than 2-fold increase in mRNA expression in tumor v.s. normal. On the other hand, microarray analysis was also performed using TCam-2 cells, which were treated with or without DHT (10^−7^M for 4 hr). 1,500 genes (light gray circle) showed a more than 2-fold decrease in DHT treated TCam-2 cells. **B.** TCam-2 cells were transfected with siAR for 3 days. 10^−7^M DHT or EtOH were added to the cultured medium for 24 hr until cell extraction. RNA expression of TPH1 in the TCam-2 cells was measured using quantitative RT-PCR. n = 2, *, *p* < 0.05.

To identify genes that are associated with SE progression and androgen/AR signal, we first compared gene expression profiles of cancer tissues from SE patients and matched normal adjacent tissues (Supplemental Table 1). A Bioanalyzer (Agilent Technologies) was used to confirm the quality of RNA extracted from human samples. A total of 925 genes among the tested 50599 genes exhibited a more than 2-fold increase in mRNA expression in SE cancer tissues compared with normal adjacent tissues. These 925 genes were categorized using gene ontology (GO) analysis and many of the genes in the biological process categories were annotated with GO term metabolic process (data not shown). Next, we performed DNA microarray analysis using TCam-2 cells treated with and without DHT. Nineteen genes among the 925 up-regulated genes in SE patients exhibited a more than 2-fold decrease in TCam-2 cells treated with DHT (Figure 4A and Supplemental Table 2 and 3). Among these 19 genes, we focused on TPH1, which is associated with the metabolism of serotonin, because dysregulation of serotonin metabolism is known to be associated with cancer progression [17]. Quantitative RT-PCR revealed that the expression of TPH1 was reduced by DHT treatment and this reduction was not observed in AR knockdown cells (Figure 4B). These results suggested that the up-regulation of TPH1 through suppression of androgen/AR signal may be associated with SE progression.

### Knockdown of TPH1 decreased serotonin level and suppressed cell growth in SE cells

**Figure 5:**
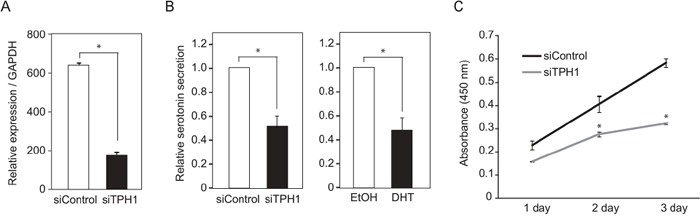
Suppression of TCam-2 cell proliferation by TPH1 knockdown **A.** Confirmation of TPH1 knockdown efficiency by quantitative RT-PCR. TPH1 siRNA or control siRNA was transfected to TCam-2 cells for 3 days. The mRNA levels of TPH1 were normalized to GAPDH. n = 2, *, *p* < 0.01. **B.** Serotonin levels in TCam-2 cell culture supernatants were exarmined by ELISA. For TPH1 knockdown, siRNA was transfected to TCam-2 cells for 3 days. DHT was added to the cultured medium at a concentration of 10^−7^ M for 24 hrs. **C.** TCam-2 cells were transfected with control siRNA or TPH1 siRNA for 3 days. After that, the number of living cells was examined by measurement of absorbance (450 nm). Data are presented as mean ± s.d. (n=3), *, *p* < 0.01.

TPH1 is an isoenzyme of tryptophan hydroxylases which catalyze monooxygenation of tryptophan to 5-hydroxytryptophan (5-HTP) [18]. 5-HTP is subsequently decarboxylated to form the neurotransmitter serotonin (5-hydroxytryptamine/5-HT). To investigate whether TPH1 could function as serotonin synthesis enzyme in TCam-2 cells, we examined serotonin synthesis in TPH1 knockdown TCam-2 cells. The mRNA expression of TPH1 in TCam-2 cells exhibited 0.3-fold decrease by the knockdown of TPH1 (Figure 5A). Under this condition, serotonin level in culture medium from TCam-2 cells was reduced (Figure 5B). Serotonin level in TCam-2 cell culture supernatant was also decreased by DHT treatment (Figure 5B). Furthermore, cell growth of TCam-2 cells was also suppressed by knockdown of TPH1 (Figure 5C). These results suggested that TPH1 could function as serotonin synthesis enzyme in SE cells and then promote cancer cell growth.

### Inhibition of signal downstream of serotonin receptor resulted in SE cell growth suppression

**Figure 6:**
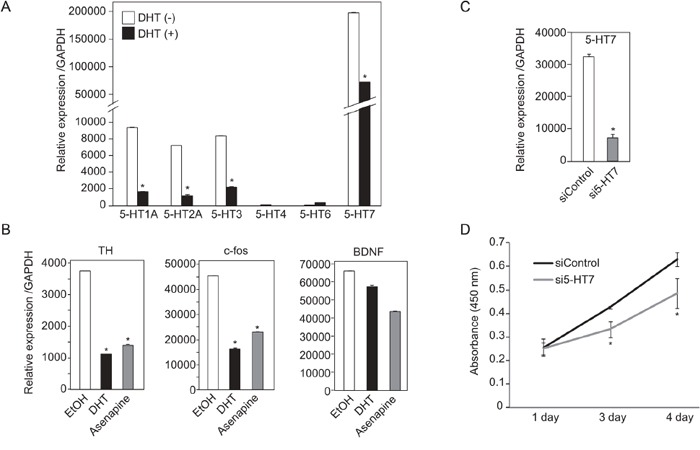
Expression and functions of 5-HTs in TCam-2 cells **A.** mRNA expression of six type of 5-HTs in TCam-2 were examined by quantitative RT-PCR. 10^−7^ M DHT or EtOH were added to the cultured medium for 24 hrs until cell extraction. mRNA levels of 5-HTs were normalized to GAPDH. n = 2, *, *p* < 0.01. **B.** mRNA expression of downstream genes of 5-HTs in TCam-2 cells were examined by quantitative RT-PCR. DHT (10–7 M) or Asenapine maleate (10–9 M) was added to the cultured medium for 24hr. **C.** Confirmation of 5-HT7 knockdown efficiency by quantitative RT-PCR. 5-HT7 siRNA or control siRNA was transfected to TCam-2 cells for 3 days. **D.** TCam-2 cells were trasnfected with control siRNA or 5-HT7 siRNA for 3 days. After that, the number of living cells was measured by determination of absorbance (450 nm). Data are presented as mean ± s.d. (n=3), *, *p* < 0.01.

Serotonin is a ligand for serotonin receptors (5-HTs) which are located in cell membrane and activate the downstream signals. Fourteen of 5-HTs have been known and they were classified to seven families [19] [20]. Each of 5-HTs has different functions and their expression shows different distribution [20]. To investigate the role of serotonin in SE cells, we examined mRNA expression level of six of 5-HTs (5-HT1A, 5-HT2A, 5-HT3, 5-HT4, 5-HT-6 and 5-HT7) in TCam-2 cells using quantitative RT-PCR. As the results, four of 5-HTs (5-HT7, 5-HT1A, 5-HT2A and 5-HT3) were highly expressed in TCam-2 cells. These four 5-HTs were decreased by DHT treatment (Figure 6A). Meanwhile, 5-HT4 and 5-HT6 were barely expressed in TCam-2 cells (Figure 6A). It has been known that activation of 5-HTs resulted in activation of transcriptional factor, cAMP response element-binding protein (CREB), in brain or several organs [21]. Many reports show that CREB binds to certain DNA sequences called *cAMP response elements* (CRE), thereby increasing or decreasing the transcription of the downstream genes [22]. We found that three CREB downstream genes, *Tyrosine hydroxylase (TH)*, *FBJ murine osteosarcoma viral oncogene homolog (c-fos)* and *brain-derived neurotrophic factor (BDNF)*, were expressed in TCam-2 cells (Figure 6B). Transcripts of *TH* or *c-fos* were decreased by DHT (Figure 6B). Asenapine maleate, which could inhibit 5-HT1A, 1B, 2A, 2B, 2C, 5A, 6 and 7 [23], also decreased TH or c-fos (Figure 6B). Finally, we examined effects of 5-HT7 knockdown on cell growth of TCam-2. The mRNA expression of 5-HT7 in TCam-2 cells exhibited 0.2-fold decrease by the knockdown of 5-HT7 (Figure 6C). TCam-2 cell growth was suppressed by 5-HT7 knockdown in TCam-2 cells (Figure 6D). These results suggested that serotonin signaling pathway could function in SE cells and promoted cell growth of SE. Considering together that DHT had inhibitory effects to the serotonin synthesis pathway in SE cells (Figure 5B), DHT may suppress SE cell growth by inhibition of TPH1-mediated serotonin synthesis pathway.

## DISCUSSION

In this report, we demonstrated that activation of androgen/AR signal had a suppressive effect of SE cell growth through the down-regulation of TPH1. Furthermore, DHT treatment reduced the expression of both serotonin and its receptors in SE cells. These results suggest that activation of androgen/AR signal and suppression of TPH1-serotonin pathway may be useful to treat SEs.

Dysregulation in normal organogenesis is associated with initiation and progression of cancer [24, 25]. Because the androgen/AR signal has a critical role in normal organogenesis and spermatogenesis in testes [5], it is reasonable to think that insufficiency of the signal may lead to initiation of testicular cancer. In fact, analysis of AR-knockout (KO) mice indicated that insufficiency of androgen/AR signaling in the testes results in accumulation of immature germ cells [26]. Previous reports have suggested that activation of androgen/AR signaling has a positive effect on the initiation and progression of various cancer cells, including prostate cancer, renal cell carcinoma [27], bladder cancer [28], and hepatocellular carcinoma [29]. On the other hand, androgen/AR signaling also has a suppressive effect in some cell types [30]. In this report, we clearly showed that androgen/AR signaling had a suppressive effect on SE cell growth and that suppression of androgen/AR signaling induced cell growth of SE *in vivo*. These results suggest that inactivation of androgen/AR signaling may lead to the progression of SEs and reactivation of androgen/AR signaling may inhibit SEs progression. According to these results, we hypothesize that supplementation of testosterone might be used as a tool to prevent the recurrence of SEs after conventional therapies such as the cisplatin-based chemotherapy.

TPH1 is a rate-limiting enzyme in the biosynthesis of serotonin, a monoamine neurotransmitter that has various well-characterized functions in the central nervous system (CNS). Previous reports have also described the functions of serotonin in various cancer growth and progression pathways. Serotonin induces the growth of cholangiocarcinoma [31], colon cancer [32], hepatocellular carcinoma [33], and pancreatic cancer cells through the serotonin receptor. Recently, several reports have suggested that TPH1 also has a role in cancer progression [17]. Immunohistochemistry analysis of breast cancer tissue using anti-TPH1 antibodies indicated that aberrant expression of TPH1 is associated with the progression of breast cancer. Additionally, in the current report, we showed that knockdown of TPH1 expression suppresses SE cell growth. Immunohistochemistry of human SE tissue using anti-TPH1 antibody may provide further evidence of the association between aberrant expression of TPH1 and SE progression.

In normal tissues, promoter analysis of TPH1 suggested that TPH1 expression levels are regulated at the transcriptional level. NF-Y and Sp1, ligand-independent transcription factors, bind to the promoter of *TPH1* and activate *TPH1* transcription [34]. However, the mechanism underlying the regulation of TPH1 expression in cancer remains unclear. In this report, we showed that activation of androgen/AR signaling repressed TPH1 expression in SE cells. Our results revealed that knockdown of AR expression abolished this effect, supporting the hypothesis that the AR is involved in the regulation of TPH1. Although the effects of the AR on gene repression are largely unknown, a previous report suggested that the repressor element (RE)-1 silencing transcription factor (REST) mediates the effects of AR on suppression of brain-derived neurotrophic factor (BDNF) in prostate cancer cells [35]. Further studies are needed to analyze the role of REST in the regulation of the *TPH1* gene.

In conclusion, we showed in this study that androgen/AR signal had a negative effect of the progression of SEs. Although further studies are required, these results suggest the possibility that androgen/AR signaling pathway may have a potential to become a therapeutic candidate for SEs.

## MATERIALS AND METHODS

### Antibodies, reagents and siRNAs

The following antibodies were used: anti-AR N-20 (sc-816, Santa Cruz) and anti-β-actin (A5441, Sigma). DHT (5a-Androstain-17b-ol-3-one) and Asenapine maleate were purchase from Sigma. siRNAs specific for TPH1 (TPH1HSS110923) and 5-HT7 (HTR7HSS105140) were purchased from Invitrogen. siRNA for AR (s1539) was purchased from Ambion. A nonspecific control siRNA pool (siControl) was purchased from Dharmacon (D-001206-13-20).

### Cell culture and transfection

TCam-2 cells were gifted from S. Nakazawa. NCCIT, NEC8, and NEC14 cell lines were purchased from ATCC. These cells were cultured in RPMI1640 (Nacalai tesque) with 10% fetal bovine serum (FBS). DHT was added to the cultured medium at a concentration of 10^−7^ for 24 hrs until cell extraction. Asenapine maleate was added to the cultured medium at a concentration of 10^−9^ M for 24 hrs until cell extraction.

For knockdown in TCam-2 cells, transfection of siRNA was performed using Lipofectamine RNAiMAX (Invitrogen) with antibiotic-free medium for more than 3 days.

### RNA extraction and quantitative RT-PCR

One microgram of total RNA was extracted from each sample using ISOGEN (NIPPON GENE) and was transcribed into first-strand cDNA using PrimeScript RT Master Mix (Takara). Real-time quantitative RT-PCR was performed using Thunderbird SYBR qPCR Mix (Toyobo) with a Thermal Dice Cycler (TaKaRa), according to the manufacturer's instructions. Primer sets for PCR were as follows: *AR* forward: 5′-TACCGCATGCACAAGTCCCG-3′, reverse: 5′-TCACTGGGTGTGGAAATAGA-3′, *TPH1* forward: 5′-CCGCAGGACCAGTTTCCATAG-3′, reverse: 5′-TCTTACCACCGGCAGATTGAGAG-3′, *GAPDH* forward: 5′-ACCACAGTCCATGCCATCAC-3′, reverse: 5′-TCCACCACCCTGTTGCTGTA-3′, *5-HT1A* forward: 5′-ACCGTCAGCTACCAAGTGAT-3′, reverse: 5′-GTTGAGCACCTGATACAGCG-3′, *5-HT2A* forward: 5′-AAAGCGGGGTTCAATGCTAC-3′, reverse: 5′-GCATAGCAGCAAGGTTTCCA-3′, *5-HT3* forward: 5′-CAGCAATCACAAGCCAAGGT-3′, reverse: 5′-TTCAGGTGAGGGTCAATGGG-3′, *5-HT4* forward: 5′-CATGGTCAACAAGCCCTACG-3′, reverse: 5′-CTTGGCTGCTTTGGTCTCTG-3′, *5-HT6* forward: 5′-AGATTCGGACTCAGACGCAG-3′, reverse: 5′-GGGTCTGGGTTCTGCTCAAT-3′, *5-HT7* forward: 5′-CACAAGTTTCCTGGCTTCCC-3′, reverse: 5′-AGATGAAGGGTCTGGCTGTC-3′, *BDNF* forward: 5′-GAGCCCTGTATCAACCCAGA-3′, reverse: 5′-TCAAATACCATGCCCCACCT-3′, *c-fos* forward: 5′- GGAGGACCTTATCTGTGCGT-3′, reverse: 5′- AAAGAGACACAGACCCAGGC-3′, *TH* forward: 5′- CTCCCCTGGTCCTGCACT-3′, reverse: 5′- GCAGCACAACCTCACCAC-3′. GAPDH was used as an internal control. We performed normalization and calculation steps as reported previously [25].

### Cell growth assay

TCam-2 cells were plated in each well of 96-well plates (5,000 or 10,000 cells per well). DHT was added to the cultured medium at a concentration of 10^−7^ M at this time (0 day). After 3 or 4 days culture, living cells were counted using a Cell Counting Kit-8 (Dojindo) and a micro plate reader (VERSA Max) [36]. When this assay was performed using knock-down cells, TCam-2 cells were transfected with siRNAs using Lipofectamine RNAiMAX for 3days and then plated in 96-well plates (0 day).

### Colony formation assay

TCam-2 cells were treated with DHT (10^−7^ M) or ethanol. The cells were then plated in each well of a 6-well plate and cultured for 2 weeks. At the end of the assay, the cells were stained with Giemsa staining, and colonies were observed by the naked eye [10].

### Tumor xenografts

Castration or sham operation was performed 5-week-old male SCID mice. On the same day, TCam-2 cells (2 × 10^7^ cells in 50 μL phosphate-buffered saline [PBS]) were subcutaneously implanted on the right dorsal gluteal region of the castrated male SCID mice (day 0). On day 45, we sacrificed the mice and excised the primary tumors (n=8). All animal procedures were approved by the Animal Care and Use Committee of Kyoto Prefectural University of Medicine before the experiment and performed in accordance with the Guidelines for Animal Care of Kyoto Prefectural University of Medicine.

### Human tumor samples

Six samples of histologically normal testis lesions and cancer lesions were obtained from surgical specimens of patients who underwent radical orchiectomy at Kyoto Prefectural University of Medicine (Table S1). RNA extraction and quantitative RT-PCR were performed as previously described [25]. A Bioanalyzer (Agilent Technologies) was used to confirm the quality of RNA extracted from human samples. The use of surgical and autopsy specimens for molecular analysis was approved by the hospital's institutional ethical committee.

### Microarray analysis

TCam-2 cells were cultured in 10^−7^M DHT or EtOH containing medium for 4 hrs. Total RNA from TCam-2 cells and human tumor samples were isolated as described above. RNA was labeled with a probe using a labeling kit (Agilent Technologies). The probe was hybridized to the microarray chip (SurePrint G3 Human Gene Expression 8×60K, Agilent Technologies). Data were analyzed using GeneSpringGX software.

### Serotonin ELISA

Serotonin ELISA was performed as described [37]. TCam-2 cells were plated at a density of 1 × 10^5^ cells/cm^2^ in 96-well plates. When this assay was performed using TPH1 knock-down cells, cells were transfected with siRNAs using Lipofectamine RNAiMAX for 3days and then plated in 96-well plates. After 24 hrs cultured medium was removed, and cells were washed and serum-free RPMI medium for 6 hrs. DHT was added to the cultured medium at a concentration of 10^−7^ M at this time. Serotonin concentration in the medium was quantified using Serotonin ELISA kit (Abnova) according to the manufacture's directions. The amount of serotonin in the medium relative to the control groups was calculated.

### Statistical analysis

Statistical analyses were carried out by *t*-test as appropriate. All data are reported as means ± SD. A *P*-value of < 0.05 was considered significant.

## SUPPLEMENTARY FIGURE AND TABLES




