# Supplementary material for: Androgen suppresses testicular cancer cell growth in vitro and in vivo
Source: Oncotarget. 2016 Apr 29;7(23):35224–32. doi: 10.18632/oncotarget.9109 (PMC5085223; doi:10.18632/oncotarget.9109)
Supplement: Supplementary file 1 [file oncotarget-07-35224-s001.pdf]

SUPPLEMENTARY FIGURE AND TABLES

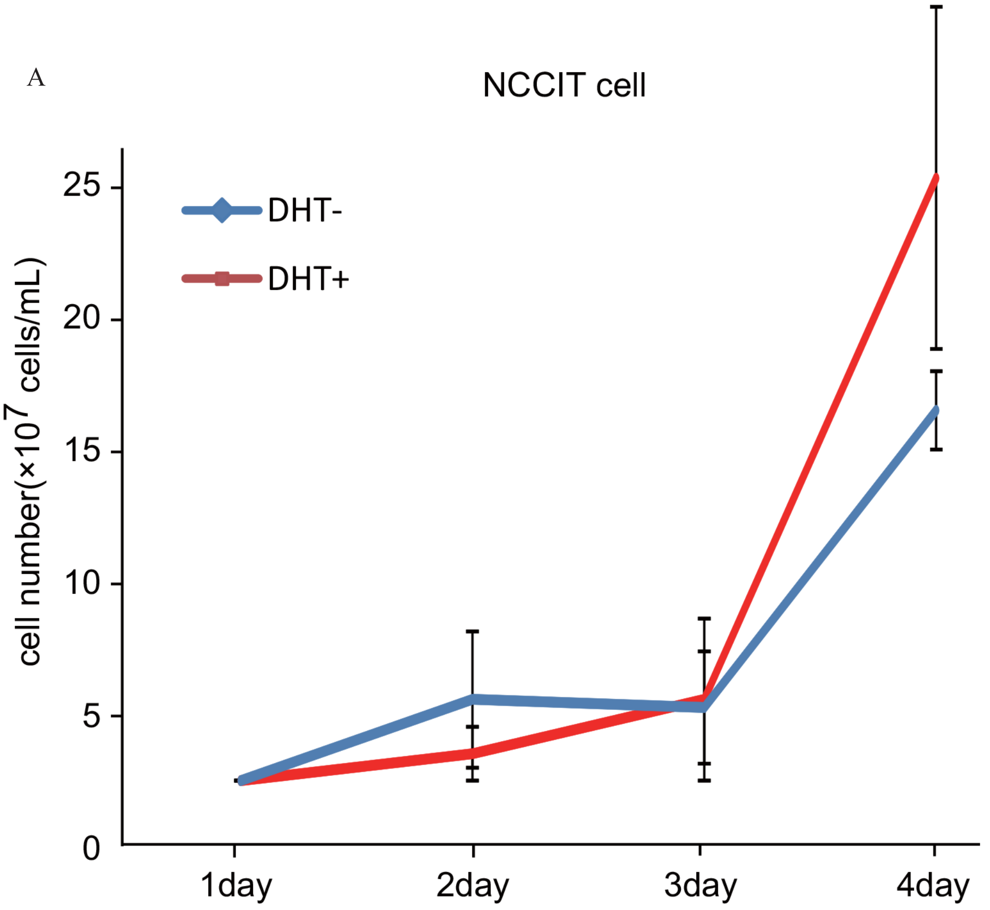

Supplementary Figure S1: Cell proliferation was not suppressed by DHT treatment in NSE cells. A. NSE cells were cultured in EtOH or DHT containing medium.growthNumber of cells was counted by the naked eye.

Supplementary Table S1: Clinicopathological features of 6 seminoma patients

sample-ID	histology	age	level of tumor markers				stage
			hCG	hCGb	LDH	AFP	
S-1	seminoma	29	8	>0.1	183	ND	I
S-2	seminoma	72	>0.4	>0.1	215	3.1	I
S-3	seminoma	44	>0.4	>0.1	166	4.4	I
S-4	seminoma	46	64	3.3	754	1.8	IIB
S-5	seminoma	46	<0.4	0.3	338	6.7	IIA
S-6	seminoma	57	<0.4	<0.1	268	2.8	IIA

Supplementary Table S2: Microarray analysis of gene expression in SE samples and TCam-2 cells

Probe Name	FC ([normal tissue] vs [cancer tissue])	FC ([TCam-2 without DHT treatment] vs [TCam-2 with DHT treatment])	Gene Symbol	Description
A_33_P3221059	-4.9043984	2.1606202		
A_19_P00323034	-4.716373	6.2507515		Homo sapiens, clone IMAGE:5528960, mRNA. [BC047326]
A_23_P150595	-4.62728	2.058645	TPH1	Homo sapiens tryptophan hydroxylase 1 (TPH1), mRNA [NM_004179]
A_33_P3227706	-4.2695093	2.463157	OR2Z1	Homo sapiens olfactory receptor, family 2, subfamily Z, member 1 (OR2Z1), mRNA [NM_001004699]
A_24_P236753	-4.2422023	2.1813443	DOC2B	Homo sapiens double C2-like domains, beta (DOC2B), mRNA [NM_003585]
A_23_P151598	-4.0110717	2.134196	CPNE6	Homo sapiens copine VI (neuronal) (CPNE6), transcript variant 2, mRNA [NM_006032]
A_33_P3262083	-3.7374272	2.055334	LOC100126784	Homo sapiens uncharacterized LOC100126784 (LOC100126784), long non-coding RNA [NR_015384]
A_21_P0007333	-3.7079165	3.9919052		
A_19_P00322673	-3.57439	2.299535		
A_21_P0005334	-3.5739617	2.4692082		
A_21_P0002562	-3.3141341	3.1461248		
A_33_P3358469	-3.0831363	2.3644578	GLI2	Homo sapiens GLI family zinc finger 2 (GLI2), mRNA [NM_005270]
A_21_P0006021	-2.888067	5.162127		
A_21_P0012123	-2.8870552	2.394399	LOC100505663	PREDICTED: Homo sapiens uncharacterized LOC100505663 (LOC100505663), transcript variant X2, misc_RNA [XR_109600]
A_21_P0008582	-2.655965	2.1198006		
A_33_P3383561	-2.638577	2.0946505	POLR2F	polymerase (RNA) II (DNA directed) polypeptide F [Source:HGNC Symbol;Acc:9193] [ENST00000443002]
A_21_P0012973	-2.4357421	6.7362804		AGENCOURT_13890273 NIH_MGC_147 Homo sapiens cDNA clone IMAGE:30346469 5', mRNA sequence [CD110012]
A_33_P3282733	-2.3486636	2.3506603	LOC100131395	PREDICTED: Homo sapiens uncharacterized LOC100131395 (LOC100131395), misc_RNA [XR_110093]
A_33_P3403102	-2.2755005	3.519007	PRR18	Homo sapiens proline rich 18 (PRR18), mRNA [NM_175922]

This table shows 19 genes, which increased in SE tumor samples and decreased by DHT treatment in TCam-2 cells.

Supplementary Table S3: Microarray analysis of gene expression in SE samples. The genes, which showed increase in tumor tissues compared to in normal tissues, are showed in this table.

See Supplementary File 1
