# Supplementary material for: Androgen suppresses testicular cancer cell growth in vitro and in vivo
Source: Oncotarget. 2016 Apr 29;7(23):35224–32. doi: 10.18632/oncotarget.9109 (PMC5085223; doi:10.18632/oncotarget.9109)
Supplement: Supplementary file 2 [file oncotarget-07-35224-s002.docx]

Supplementary Table3

| ProbeName | FC ([normal tissue] vs [cancer tissue]) | GeneSymbol | Description |
| --- | --- | --- | --- |
| A_23_P164596 | -9.962601 | SIGLEC12 | Homo sapiens sialic acid binding Ig-like lectin 12 (gene/pseudogene) (SIGLEC12), transcript variant 1, mRNA [NM_053003] |
| A_24_P346855 | -9.33505 | MKI67 | Homo sapiens marker of proliferation Ki-67 (MKI67), transcript variant 1, mRNA [NM_002417] |
| A_21_P0000460 | -9.130639 | SNORD121A | Homo sapiens small nucleolar RNA, C/D box 121A (SNORD121A), small nucleolar RNA [NR_003685] |
| A_21_P0000395 | -9.082043 | SNORD111 | Homo sapiens small nucleolar RNA, C/D box 111 (SNORD111), small nucleolar RNA [NR_003079] |
| A_23_P19030 | -8.26431 | ARSI | Homo sapiens arylsulfatase family, member I (ARSI), mRNA [NM_001012301] |
| A_21_P0007329 | -7.352383 |  | PREDICTED: Homo sapiens uncharacterized LOC101928260 (LOC101928260), misc_RNA [XR_242859] |
| A_19_P00322705 | -7.342304 | MIAT | Homo sapiens myocardial infarction associated transcript (non-protein coding) (MIAT), transcript variant 1, long non-coding RNA [NR_003491] |
| A_21_P0005041 | -7.216712 |  |  |
| A_21_P0000514 | -7.0523067 | VTRNA1-2 | Homo sapiens vault RNA 1-2 (VTRNA1-2), vault RNA [NR_026704] |
| A_33_P3403075 | -7.039517 | PRR11 | Homo sapiens proline rich 11 (PRR11), mRNA [NM_018304] |
| A_32_P229132 | -6.8821955 | FMN2 | Homo sapiens formin 2 (FMN2), mRNA [NM_020066] |
| A_32_P523096 | -6.7270937 | FOXD2-AS1 | Homo sapiens FOXD2 antisense RNA 1 (head to head) (FOXD2-AS1), long non-coding RNA [NR_026878] |
| A_32_P221799 | -6.5416327 | HIST1H2AM | Homo sapiens histone cluster 1, H2am (HIST1H2AM), mRNA [NM_003514] |
| A_33_P3374205 | -6.4748034 | MKI67 | Homo sapiens marker of proliferation Ki-67 (MKI67), transcript variant 1, mRNA [NM_002417] |
| A_23_P55477 | -6.267992 | ADORA2B | Homo sapiens adenosine A2b receptor (ADORA2B), mRNA [NM_000676] |
| A_21_P0009332 | -6.2674947 |  |  |
| A_33_P3385912 | -6.266221 |  |  |
| A_33_P3374210 | -6.22606 | MKI67 | Homo sapiens marker of proliferation Ki-67 (MKI67), transcript variant 1, mRNA [NM_002417] |
| A_23_P146146 | -6.2158175 | ATP6V0D2 | Homo sapiens ATPase, H+ transporting, lysosomal 38kDa, V0 subunit d2 (ATP6V0D2), mRNA [NM_152565] |
| A_24_P177553 | -6.177534 |  | AGENCOURT_10615922 NIH_MGC_141 Homo sapiens cDNA clone IMAGE:6744194 5', mRNA sequence [BU963192] |
| A_33_P3315564 | -6.1673875 | SULT1C3 | Homo sapiens sulfotransferase family, cytosolic, 1C, member 3 (SULT1C3), mRNA [NM_001008743] |
| A_23_P363174 | -6.1437383 | HIST1H2AL | Homo sapiens histone cluster 1, H2al (HIST1H2AL), mRNA [NM_003511] |
| A_33_P3232086 | -6.12761 | CD177 | CD177 molecule [Source:HGNC Symbol;Acc:30072] [ENST00000378009] |
| A_21_P0005010 | -6.02291 |  |  |
| A_33_P3256490 | -5.8566146 | ZYG11A | Homo sapiens zyg-11 family member A, cell cycle regulator (ZYG11A), mRNA [NM_001004339] |
| A_19_P00321061 | -5.853976 | LOC100499221 | Homo sapiens cDNA FLJ45109 fis, clone BRAWH3034097. [AK127052] |
| A_33_P3634554 | -5.8321557 |  | Q6AZG4_XENLA (Q6AZG4) LOC446243 protein (Fragment), partial (19%) [THC2601422] |
| A_21_P0013766 | -5.727923 |  |  |
| A_32_P69802 | -5.7070394 | SLCO1A2 | Homo sapiens solute carrier organic anion transporter family, member 1A2 (SLCO1A2), transcript variant 1, mRNA [NM_134431] |
| A_24_P297539 | -5.679934 | UBE2C | Homo sapiens ubiquitin-conjugating enzyme E2C (UBE2C), transcript variant 4, mRNA [NM_181801] |
| A_21_P0010164 | -5.6419835 |  |  |
| A_21_P0003263 | -5.634308 | LINC00578 | Homo sapiens long intergenic non-protein coding RNA 578 (LINC00578), long non-coding RNA [NR_047568] |
| A_21_P0010634 | -5.5686655 |  |  |
| A_33_P3373771 | -5.56798 |  |  |
| A_33_P3376116 | -5.5612826 | SPC24 | Homo sapiens SPC24, NDC80 kinetochore complex component (SPC24), mRNA [NM_182513] |
| A_23_P216355 | -5.5430427 | TONSL | Homo sapiens tonsoku-like, DNA repair protein (TONSL), mRNA [NM_013432] |
| A_33_P3329552 | -5.528412 |  |  |
| A_21_P0006009 | -5.5181475 |  |  |
| A_33_P3418185 | -5.4753833 | LYNX1 | Homo sapiens Ly6/neurotoxin 1 (LYNX1), transcript variant 1, mRNA [NM_023946] |
| A_33_P3405504 | -5.472061 |  |  |
| A_21_P0005676 | -5.436626 |  |  |
| A_21_P0010302 | -5.4311996 |  | UI-E-CQ1-agc-p-01-0-UI.s1 UI-E-CQ1 Homo sapiens cDNA clone UI-E-CQ1-agc-p-01-0-UI 3', mRNA sequence [BM671314] |
| A_21_P0001474 | -5.424531 |  |  |
| A_33_P3421438 | -5.4009333 |  |  |
| A_21_P0006959 | -5.375357 |  |  |
| A_23_P83498 | -5.369084 | IGF2BP1 | Homo sapiens insulin-like growth factor 2 mRNA binding protein 1 (IGF2BP1), transcript variant 1, mRNA [NM_006546] |
| A_21_P0000371 | -5.366698 | SNORD7 | Homo sapiens small nucleolar RNA, C/D box 7 (SNORD7), small nucleolar RNA [NR_003037] |
| A_21_P0000282 | -5.3638725 | SNORD56 | Homo sapiens small nucleolar RNA, C/D box 56 (SNORD56), small nucleolar RNA [NR_002739] |
| A_21_P0000484 | -5.3299055 | SNORD76 | Homo sapiens small nucleolar RNA, C/D box 76 (SNORD76), small nucleolar RNA [NR_003942] |
| A_33_P3248245 | -5.2571692 |  | fs26e07.y1 Human Lens cDNA (Normalized): fs Homo sapiens cDNA clone fs26e07 5', mRNA sequence [CD675678] |
| A_19_P00316370 | -5.24418 | LOC100506178 | Homo sapiens uncharacterized LOC100506178 (LOC100506178), long non-coding RNA [NR_038393] |
| A_33_P3324750 | -5.220292 | SRRM2-AS1 | SRRM2 antisense RNA 1 [Source:HGNC Symbol;Acc:44162] [ENST00000382313] |
| A_33_P3319585 | -5.1809244 | FIGNL2 | Homo sapiens fidgetin-like 2 (FIGNL2), mRNA [NM_001013690] |
| A_21_P0000498 | -5.1031227 | SNORD1C | Homo sapiens small nucleolar RNA, C/D box 1C (SNORD1C), small nucleolar RNA [NR_004397] |
| A_24_P100517 | -5.0913734 | SAPCD2 | Homo sapiens suppressor APC domain containing 2 (SAPCD2), mRNA [NM_178448] |
| A_33_P3340828 | -5.0703335 |  |  |
| A_33_P3214422 | -5.041487 | ANP32A | acidic (leucine-rich) nuclear phosphoprotein 32 family, member A [Source:HGNC Symbol;Acc:13233] [ENST00000483551] |
| A_19_P00806193 | -5.038154 |  |  |
| A_33_P3230364 | -5.0350614 | OR52E2 | Homo sapiens olfactory receptor, family 52, subfamily E, member 2 (OR52E2), mRNA [NM_001005164] |
| A_33_P3336148 | -5.0137606 |  |  |
| A_33_P3351166 | -4.994929 |  |  |
| A_33_P3214066 | -4.994511 | CASC14 | Homo sapiens cancer susceptibility candidate 14 (non-protein coding) (CASC14), long non-coding RNA [NR_034143] |
| A_21_P0000209 | -4.9548144 | SNORD4B | Homo sapiens small nucleolar RNA, C/D box 4B (SNORD4B), small nucleolar RNA [NR_000009] |
| A_33_P3578325 | -4.9426804 | SNORD15A | Homo sapiens small nucleolar RNA, C/D box 15A (SNORD15A), small nucleolar RNA [NR_000005] |
| A_33_P3238920 | -4.928781 | INPP5F | Homo sapiens inositol polyphosphate-5-phosphatase F (INPP5F), transcript variant 3, mRNA [NM_001243195] |
| A_21_P0000385 | -4.9067683 | SNORD88C | Homo sapiens small nucleolar RNA, C/D box 88C (SNORD88C), small nucleolar RNA [NR_003069] |
| A_33_P3221059 | -4.9043984 |  |  |
| A_33_P3396578 | -4.8987217 | GRK5 | Homo sapiens G protein-coupled receptor kinase 5, mRNA (cDNA clone IMAGE:3912936), with apparent retained intron. [BC018116] |
| A_33_P3293082 | -4.875816 | LOC392364 | Homo sapiens nuclear pore associated protein 1 pseudogene (LOC392364), non-coding RNA [NR_040117] |
| A_21_P0006352 | -4.8592353 |  |  |
| A_21_P0011435 | -4.8507867 | RHPN2 | Homo sapiens rhophilin, Rho GTPase binding protein 2 (RHPN2), mRNA [NM_033103] |
| A_33_P3398597 | -4.850695 | EPS8L1 | Homo sapiens EPS8-like 1 (EPS8L1), transcript variant 1, mRNA [NM_133180] |
| A_33_P3367731 | -4.837114 | SLC24A2 | Homo sapiens solute carrier family 24 (sodium/potassium/calcium exchanger), member 2 (SLC24A2), transcript variant 1, mRNA [NM_020344] |
| A_19_P00326132 | -4.8251014 | TSIX | Homo sapiens TSIX transcript, XIST antisense RNA (TSIX), antisense RNA [NR_003255] |
| A_23_P258493 | -4.814741 | LMNB1 | Homo sapiens lamin B1 (LMNB1), transcript variant 1, mRNA [NM_005573] |
| A_23_P374082 | -4.781786 | ADAM19 | Homo sapiens ADAM metallopeptidase domain 19 (ADAM19), mRNA [NM_033274] |
| A_23_P320858 | -4.778496 |  | Homo sapiens chromosome 15 open reading frame 49, mRNA (cDNA clone MGC:95352 IMAGE:7216891), complete cds. [BC069077] |
| A_21_P0001196 | -4.7722154 |  |  |
| A_33_P3324934 | -4.76196 | CDH26 | cadherin 26 [Source:HGNC Symbol;Acc:15902] [ENST00000244047] |
| A_21_P0000393 | -4.759137 | SNORD99 | Homo sapiens small nucleolar RNA, C/D box 99 (SNORD99), small nucleolar RNA [NR_003077] |
| A_19_P00323034 | -4.716373 |  | Homo sapiens, clone IMAGE:5528960, mRNA. [BC047326] |
| A_21_P0001609 | -4.711298 |  |  |
| A_23_P128759 | -4.706856 | SERPINA10 | Homo sapiens serpin peptidase inhibitor, clade A (alpha-1 antiproteinase, antitrypsin), member 10 (SERPINA10), transcript variant 1, mRNA [NM_016186] |
| A_21_P0013544 | -4.6887 | LOC101929127 | PREDICTED: Homo sapiens uncharacterized LOC101929127 (LOC101929127), transcript variant X1, misc_RNA [XR_242517] |
| A_21_P0006285 | -4.6834316 |  |  |
| A_33_P3287584 | -4.660654 | PPP1R18 | Homo sapiens protein phosphatase 1, regulatory subunit 18 (PPP1R18), transcript variant 1, mRNA [NM_133471] |
| A_33_P3348569 | -4.641367 | OR9G4 | Homo sapiens olfactory receptor, family 9, subfamily G, member 4 (OR9G4), mRNA [NM_001005284] |
| A_21_P0004428 | -4.6319346 |  |  |
| A_23_P150595 | -4.62728 | TPH1 | Homo sapiens tryptophan hydroxylase 1 (TPH1), mRNA [NM_004179] |
| A_23_P345707 | -4.6248784 | TICRR | Homo sapiens TOPBP1-interacting checkpoint and replication regulator (TICRR), mRNA [NM_152259] |
| A_19_P00322571 | -4.621432 | MIAT | Homo sapiens myocardial infarction associated transcript (non-protein coding) (MIAT), transcript variant 2, long non-coding RNA [NR_033319] |
| A_21_P0002838 | -4.619532 |  | DB029717 TESTI2 Homo sapiens cDNA clone TESTI2013026 5', mRNA sequence [DB029717] |
| A_21_P0008738 | -4.607778 |  |  |
| A_33_P3390471 | -4.5951943 | TFAP2A-AS1 | Homo sapiens, clone IMAGE:3863392, mRNA. [BC012200] |
| A_33_P3289960 | -4.5840034 | LINC00222 | Homo sapiens long intergenic non-protein coding RNA 222 (LINC00222), long non-coding RNA [NR_033376] |
| A_21_P0006030 | -4.5783496 |  |  |
| A_33_P3223577 | -4.57541 |  | Q2J851_FRASC (Q2J851) ATPases involved in chromosome partitioning-like, partial (5%) [THC2515563] |
| A_23_P89380 | -4.5527987 | SLC4A1 | Homo sapiens solute carrier family 4 (anion exchanger), member 1 (SLC4A1), mRNA [NM_000342] |
| A_33_P3221084 | -4.5510306 | LTBP3 | latent transforming growth factor beta binding protein 3 [Source:HGNC Symbol;Acc:6716] [ENST00000525443] |
| A_33_P3384902 | -4.5367584 | OR6X1 | Homo sapiens olfactory receptor, family 6, subfamily X, member 1 (OR6X1), mRNA [NM_001005188] |
| A_33_P3230676 | -4.5341463 |  |  |
| A_21_P0006973 | -4.5338745 |  |  |
| A_21_P0014764 | -4.5319934 |  | Uncharacterized protein; Zinc finger domain-related protein TSRM [Source:UniProtKB/TrEMBL;Acc:Q5XPU7] [ENST00000433518] |
| A_33_P3392374 | -4.5111246 | TTBK1 | Homo sapiens tau tubulin kinase 1 (TTBK1), mRNA [NM_032538] |
| A_21_P0000376 | -4.504777 | SNORD32B | Homo sapiens small nucleolar RNA, C/D box 32B (SNORD32B), small nucleolar RNA [NR_003049] |
| A_23_P46829 | -4.5026736 | FGF8 | Homo sapiens fibroblast growth factor 8 (androgen-induced) (FGF8), transcript variant F, mRNA [NM_033163] |
| A_33_P3315325 | -4.4962707 | ANKRD46 | Homo sapiens ankyrin repeat domain 46 (ANKRD46), transcript variant 4, mRNA [NM_001270379] |
| A_23_P429363 | -4.476891 | FGF17 | Homo sapiens fibroblast growth factor 17 (FGF17), mRNA [NM_003867] |
| A_24_P203689 | -4.4699607 | KIF7 | Homo sapiens kinesin family member 7 (KIF7), mRNA [NM_198525] |
| A_21_P0000289 | -4.458478 | SNORD45A | Homo sapiens small nucleolar RNA, C/D box 45A (SNORD45A), small nucleolar RNA [NR_002749] |
| A_21_P0010292 | -4.4542856 |  |  |
| A_21_P0012536 | -4.4418383 |  |  |
| A_21_P0006530 | -4.441036 |  |  |
| A_32_P152696 | -4.439656 |  |  |
| A_32_P12104 | -4.4240365 | ANAPC1 | Homo sapiens anaphase promoting complex subunit 1 (ANAPC1), mRNA [NM_022662] |
| A_33_P3260667 | -4.4239473 | OR2T34 | Homo sapiens olfactory receptor, family 2, subfamily T, member 34 (OR2T34), mRNA [NM_001001821] |
| A_33_P3338937 | -4.41963 |  |  |
| A_33_P3324383 | -4.413534 | FIGNL2 | Homo sapiens fidgetin-like 2 (FIGNL2), mRNA [NM_001013690] |
| A_21_P0000050 | -4.3973217 | DISC1 | Homo sapiens disrupted in schizophrenia 1 (DISC1), transcript variant l, mRNA [NM_001164549] |
| A_21_P0000210 | -4.392997 | SNORD4A | Homo sapiens small nucleolar RNA, C/D box 4A (SNORD4A), small nucleolar RNA [NR_000010] |
| A_21_P0007523 | -4.390895 | DDX11-AS1 | Homo sapiens DDX11 antisense RNA 1 (DDX11-AS1), long non-coding RNA [NR_038927] |
| A_21_P0000469 | -4.3898206 | SNORD11B | Homo sapiens small nucleolar RNA, C/D box 11B (SNORD11B), small nucleolar RNA [NR_003694] |
| A_33_P3376971 | -4.375003 | CHAC1 | Homo sapiens ChaC, cation transport regulator homolog 1 (E. coli) (CHAC1), transcript variant 1, mRNA [NM_024111] |
| A_33_P3296193 | -4.372025 | PSIMCT-1 | Homo sapiens malignant T cell amplified sequence 1 pseudogene (PSIMCT-1), non-coding RNA [NR_003677] |
| A_21_P0002479 | -4.364309 |  |  |
| A_24_P912366 | -4.3635983 | FAM160A1 | Homo sapiens family with sequence similarity 160, member A1 (FAM160A1), mRNA [NM_001109977] |
| A_33_P3329063 | -4.3604813 | NCR1 | Homo sapiens natural cytotoxicity triggering receptor 1 (NCR1), transcript variant 1, mRNA [NM_004829] |
| A_21_P0001754 | -4.3601766 |  |  |
| A_21_P0011344 | -4.3517046 | RHPN2 | Homo sapiens rhophilin, Rho GTPase binding protein 2 (RHPN2), mRNA [NM_033103] |
| A_21_P0008466 | -4.3287935 |  |  |
| A_33_P3355508 | -4.323714 | FOXL2 | Homo sapiens forkhead box L2 (FOXL2), mRNA [NM_023067] |
| A_23_P160017 | -4.320522 | TTTY11 | Homo sapiens testis-specific transcript, Y-linked 11 (non-protein coding) (TTTY11), long non-coding RNA [NR_001548] |
| A_21_P0000032 | -4.316419 | KIRREL3 | Homo sapiens kin of IRRE like 3 (Drosophila) (KIRREL3), transcript variant 2, mRNA [NM_001161707] |
| A_21_P0000394 | -4.310969 | SNORD110 | Homo sapiens small nucleolar RNA, C/D box 110 (SNORD110), small nucleolar RNA [NR_003078] |
| A_33_P3286873 | -4.2877035 | CD247 | CD247 molecule [Source:HGNC Symbol;Acc:1677] [ENST00000483825] |
| A_33_P3293469 | -4.2714868 | LOC100128105 | |
| A_33_P3227706 | -4.2695093 | OR2Z1 | Homo sapiens olfactory receptor, family 2, subfamily Z, member 1 (OR2Z1), mRNA [NM_001004699] |
| A_19_P00804070 | -4.2692137 |  |  |
| A_21_P0005352 | -4.2616005 |  |  |
| A_33_P3241299 | -4.2578683 | VPS9D1-AS1 | Homo sapiens VPS9D1 antisense RNA 1 (VPS9D1-AS1), long non-coding RNA [NR_036480] |
| A_33_P3281537 | -4.2566466 | SLC27A3 | PREDICTED: Homo sapiens solute carrier family 27 (fatty acid transporter), member 3 (SLC27A3), transcript variant X2, mRNA [XM_005244852] |
| A_33_P3390264 | -4.2554636 | OR10W1 | Homo sapiens olfactory receptor, family 10, subfamily W, member 1 (OR10W1), mRNA [NM_207374] |
| A_21_P0002514 | -4.254813 |  | 603051348F1 NIH_MGC_116 Homo sapiens cDNA clone IMAGE:5191547 5', mRNA sequence [BI765130] |
| A_24_P236753 | -4.2422023 | DOC2B | Homo sapiens double C2-like domains, beta (DOC2B), mRNA [NM_003585] |
| A_21_P0000187 | -4.2359776 | LOC100287036 | Homo sapiens uncharacterized LOC100287036 (LOC100287036), mRNA [NM_001242885] |
| A_24_P231026 | -4.2288294 | SCN8A | Homo sapiens sodium channel, voltage gated, type VIII, alpha subunit (SCN8A), transcript variant 1, mRNA [NM_014191] |
| A_21_P0006120 | -4.2158847 |  |  |
| A_21_P0000259 | -4.209251 | SNORD25 | Homo sapiens small nucleolar RNA, C/D box 25 (SNORD25), small nucleolar RNA [NR_002565] |
| A_21_P0000221 | -4.2070885 | SNORD32A | Homo sapiens small nucleolar RNA, C/D box 32A (SNORD32A), small nucleolar RNA [NR_000021] |
| A_33_P3356637 | -4.206111 |  |  |
| A_24_P75963 | -4.2029276 | DSCR4 | Homo sapiens Down syndrome critical region gene 4 (DSCR4), mRNA [NM_005867] |
| A_32_P132589 | -4.2019715 | ZYG11A | Homo sapiens zyg-11 family member A, cell cycle regulator (ZYG11A), mRNA [NM_001004339] |
| A_33_P3417422 | -4.197085 | ST8SIA6-AS1 | ST8SIA6 antisense RNA 1 [Source:HGNC Symbol;Acc:44880] [ENST00000377597] |
| A_21_P0006451 | -4.1967897 |  |  |
| A_33_P3252954 | -4.1951165 | NKD2 | naked cuticle homolog 2 (Drosophila) [Source:HGNC Symbol;Acc:17046] [ENST00000382730] |
| A_21_P0012472 | -4.1905637 |  | PREDICTED: Homo sapiens uncharacterized LOC101928992 (LOC101928992), misc_RNA [XR_241629] |
| A_33_P3409234 | -4.182763 |  | DA399191 BRTHA3 Homo sapiens cDNA clone BRTHA3001578 5', mRNA sequence [DA399191] |
| A_21_P0005025 | -4.1801405 |  |  |
| A_21_P0000689 | -4.1762567 | LINC00102 | Homo sapiens long intergenic non-protein coding RNA 102 (LINC00102), long non-coding RNA [NR_037842] |
| A_33_P3240098 | -4.1737914 |  | Homo sapiens cDNA clone IMAGE:40133626. [BC150587] |
| A_19_P00322836 | -4.161298 |  |  |
| A_33_P3393734 | -4.161237 | RPS27 | ribosomal protein S27 [Source:HGNC Symbol;Acc:10416] [ENST00000392558] |
| A_33_P3349671 | -4.159841 | LOC101927680 | PREDICTED: Homo sapiens ERV-FRD provirus ancestral Env polyprotein-like (LOC101927680), mRNA [XM_005252326] |
| A_23_P428373 | -4.158851 | REXO1L1 | PREDICTED: Homo sapiens REX1, RNA exonuclease 1 homolog (S. cerevisiae)-like 1 (REXO1L1), transcript variant X1, mRNA [XM_005251207] |
| A_33_P3237905 | -4.1325817 |  |  |
| A_33_P3312147 | -4.1319437 |  |  |
| A_21_P0000240 | -4.130868 | SNORD100 | Homo sapiens small nucleolar RNA, C/D box 100 (SNORD100), small nucleolar RNA [NR_002435] |
| A_33_P3297587 | -4.1211185 | LINC00244 | Homo sapiens long intergenic non-protein coding RNA 244 (LINC00244), long non-coding RNA [NR_024119] |
| A_33_P3260782 | -4.1152377 | RAG2 | recombination activating gene 2 [Source:HGNC Symbol;Acc:9832] [ENST00000534379] |
| A_33_P3402868 | -4.109511 | GRIN2D | Homo sapiens glutamate receptor, ionotropic, N-methyl D-aspartate 2D (GRIN2D), mRNA [NM_000836] |
| A_33_P3280916 | -4.094067 | SNHG4 | Homo sapiens small nucleolar RNA host gene 4 (non-protein coding) (SNHG4), transcript variant 1, long non-coding RNA [NR_003141] |
| A_33_P3417427 | -4.093528 | ST8SIA6-AS1 | ST8SIA6 antisense RNA 1 [Source:HGNC Symbol;Acc:44880] [ENST00000377597] |
| A_33_P3303752 | -4.0920997 |  |  |
| A_33_P3385477 | -4.091081 | ATAD3B | Homo sapiens ATPase family, AAA domain containing 3B (ATAD3B), mRNA [NM_031921] |
| A_21_P0000227 | -4.0847383 | SNORD35B | Homo sapiens small nucleolar RNA, C/D box 35B (SNORD35B), small nucleolar RNA [NR_001285] |
| A_21_P0001068 | -4.0829797 |  |  |
| A_21_P0014180 | -4.081629 | GPR146 | G protein-coupled receptor 146 [Source:HGNC Symbol;Acc:21718] [ENST00000474396] |
| A_33_P3286002 | -4.080877 | VKORC1 | Homo sapiens cDNA FLJ43630 fis, clone SPLEN2030479. [AK125618] |
| A_33_P3239322 | -4.0697346 | LOC100132474 | Homo sapiens cDNA FLJ43170 fis, clone FCBBF3004847. [AK125160] |
| A_21_P0007180 | -4.0661488 |  |  |
| A_23_P300100 | -4.0506706 | PLA2G2D | phospholipase A2, group IID [Source:HGNC Symbol;Acc:9033] [ENST00000375105] |
| A_21_P0000170 | -4.050506 | SMIM18 | Homo sapiens small integral membrane protein 18 (SMIM18), mRNA [NM_001206847] |
| A_33_P3232516 | -4.048224 | KIF18B | Homo sapiens kinesin family member 18B (KIF18B), transcript variant 1, mRNA [NM_001265577] |
| A_24_P697685 | -4.0462675 | ESYT3 | Homo sapiens extended synaptotagmin-like protein 3 (ESYT3), mRNA [NM_031913] |
| A_33_P3351609 | -4.024535 |  | Q80T73_MOUSE (Q80T73) MKIAA1930 protein (Fragment), partial (3%) [THC2555232] |
| A_33_P3209185 | -4.015142 |  |  |
| A_21_P0009943 | -4.0130033 |  |  |
| A_23_P151598 | -4.0110717 | CPNE6 | Homo sapiens copine VI (neuronal) (CPNE6), transcript variant 2, mRNA [NM_006032] |
| A_21_P0001575 | -4.0077324 |  |  |
| A_23_P88691 | -4.0028925 | CHRNA5 | Homo sapiens cholinergic receptor, nicotinic, alpha 5 (neuronal) (CHRNA5), mRNA [NM_000745] |
| A_21_P0005358 | -3.9983492 |  |  |
| A_21_P0002091 | -3.989208 |  | DA874553 PROST2 Homo sapiens cDNA clone PROST2012117 5', mRNA sequence [DA874553] |
| A_24_P16856 | -3.9871793 | AKAP17A | A kinase (PRKA) anchor protein 17A [Source:HGNC Symbol;Acc:18783] [ENST00000381261] |
| A_33_P3802966 | -3.986442 |  | Q27D47_MYCFV (Q27D47) Mammalian cell entry related, partial (3%) [THC2752020] |
| A_21_P0014840 | -3.9734297 | LOC100507630 | Homo sapiens PRO2015 mRNA, complete cds. [AF119861] |
| A_21_P0014586 | -3.9732983 | TNK2-AS1 | Homo sapiens cDNA FLJ45707 fis, clone FEHRT2001482. [AK127609] |
| A_21_P0001216 | -3.9693887 |  | PREDICTED: Homo sapiens uncharacterized LOC101929219 (LOC101929219), transcript variant X4, misc_RNA [XR_248680] |
| A_21_P0002890 | -3.967382 |  |  |
| A_33_P3294042 | -3.9659858 |  | T cell receptor beta variable 10-1(gene/pseudogene) [Source:HGNC Symbol;Acc:12177] [ENST00000390364] |
| A_21_P0009897 | -3.9570584 |  |  |
| A_23_P7976 | -3.955235 | HIST1H1E | Homo sapiens histone cluster 1, H1e (HIST1H1E), mRNA [NM_005321] |
| A_21_P0009673 | -3.9549756 |  | PREDICTED: Homo sapiens uncharacterized LOC101927522 (LOC101927522), misc_RNA [XR_243988] |
| A_33_P3218450 | -3.9427104 | CDC6 | Homo sapiens cell division cycle 6 (CDC6), mRNA [NM_001254] |
| A_23_P155931 | -3.927175 | MEPE | Homo sapiens matrix extracellular phosphoglycoprotein (MEPE), transcript variant 2, mRNA [NM_020203] |
| A_21_P0001684 | -3.9249542 |  |  |
| A_33_P3331588 | -3.9164746 | ATAD3B | Homo sapiens ATPase family, AAA domain containing 3B (ATAD3B), mRNA [NM_031921] |
| A_33_P3256287 | -3.9149513 | FAM95B1 | family with sequence similarity 95, member B1 [Source:HGNC Symbol;Acc:32318] [ENST00000455995] |
| A_23_P211326 | -3.9108922 | CECR2 | Homo sapiens cat eye syndrome chromosome region, candidate 2 (CECR2), mRNA [NM_031413] |
| A_21_P0006408 | -3.9103005 |  | PREDICTED: Homo sapiens uncharacterized LOC101928302 (LOC101928302), transcript variant X1, misc_RNA [XR_247298] |
| A_21_P0003803 | -3.9057117 |  |  |
| A_33_P3355004 | -3.8956943 | DDC | Homo sapiens dopa decarboxylase (aromatic L-amino acid decarboxylase) (DDC), transcript variant 7, mRNA [NM_001242890] |
| A_21_P0000390 | -3.8853407 | SNORD92 | Homo sapiens small nucleolar RNA, C/D box 92 (SNORD92), small nucleolar RNA [NR_003074] |
| A_33_P3240679 | -3.8830757 | BRD3 | bromodomain containing 3 [Source:HGNC Symbol;Acc:1104] [ENST00000371842] |
| A_21_P0007477 | -3.8803916 | LOC100131138 | Homo sapiens uncharacterized LOC100131138 (LOC100131138), long non-coding RNA [NR_036513] |
| A_23_P413641 | -3.8765595 | PREX1 | Homo sapiens phosphatidylinositol-3,4,5-trisphosphate-dependent Rac exchange factor 1 (PREX1), mRNA [NM_020820] |
| A_33_P3363001 | -3.8764992 |  | DA372173 BRTHA2 Homo sapiens cDNA clone BRTHA2002342 5', mRNA sequence [DA372173] |
| A_33_P3411357 | -3.8726401 | DGCR11 | Homo sapiens DiGeorge syndrome critical region gene 11 (non-protein coding) (DGCR11), long non-coding RNA [NR_024157] |
| A_21_P0007753 | -3.8672667 |  |  |
| A_33_P3229335 | -3.864486 | HIST3H2BB | Homo sapiens histone cluster 3, H2bb (HIST3H2BB), mRNA [NM_175055] |
| A_21_P0005661 | -3.8640966 |  |  |
| A_33_P3380086 | -3.8614123 | PXN | paxillin [Source:HGNC Symbol;Acc:9718] [ENST00000323871] |
| A_21_P0012324 | -3.8608623 |  | ALU1_HUMAN (P39188) Alu subfamily J sequence contamination warning entry, partial (15%) [THC2687080] |
| A_19_P00318511 | -3.849348 |  | Homo sapiens, clone IMAGE:3960940, mRNA, partial cds. [BC014369] |
| A_24_P93948 | -3.8475826 | KIF5A | kinesin family member 5A [Source:HGNC Symbol;Acc:6323] [ENST00000455537] |
| A_23_P27606 | -3.8440702 | IL27RA | Homo sapiens interleukin 27 receptor, alpha (IL27RA), mRNA [NM_004843] |
| A_33_P3284266 | -3.8309963 |  | SCYB6_HUMAN (P80162) Small inducible cytokine B6 precursor (CXCL6) (Granulocyte chemotactic protein 2) (GCP-2) (Chemokine alpha 3) (CKA-3) [Contains: Small inducible cytokine B6, N-processed variant 1; Small inducible cytokine B6, N-processed variant 2;, complete [THC2627589] |
| A_21_P0009841 | -3.8101642 |  |  |
| A_33_P3415820 | -3.804875 | THBS1 | Homo sapiens thrombospondin 1 (THBS1), mRNA [NM_003246] |
| A_21_P0010077 | -3.7911859 | LOC101929125 | PREDICTED: Homo sapiens uncharacterized LOC101929125 (LOC101929125), transcript variant X2, misc_RNA [XR_244232] |
| A_21_P0003420 | -3.7839787 |  |  |
| A_23_P111737 | -3.7826617 | RAMP3 | Homo sapiens receptor (G protein-coupled) activity modifying protein 3 (RAMP3), mRNA [NM_005856] |
| A_32_P40377 | -3.7796564 | LOC389906 | Homo sapiens zinc finger protein 839 pseudogene (LOC389906), non-coding RNA [NR_034031] |
| A_21_P0010378 | -3.7795665 |  | DB054128 TESTI2 Homo sapiens cDNA clone TESTI2044894 5', mRNA sequence [DB054128] |
| A_21_P0009682 | -3.765846 |  |  |
| A_33_P3363655 | -3.7644694 | ANAPC1 | Homo sapiens anaphase promoting complex subunit 1 (ANAPC1), mRNA [NM_022662] |
| A_21_P0002171 | -3.7596495 |  |  |
| A_33_P3352223 | -3.757934 | CSRP1 | cysteine and glycine-rich protein 1 [Source:HGNC Symbol;Acc:2469] [ENST00000533402] |
| A_33_P3337584 | -3.7496412 | LOC283482 | PREDICTED: Homo sapiens uncharacterized LOC283482 (LOC283482), misc_RNA [XR_247950] |
| A_24_P392151 | -3.7439182 | C11orf86 | Homo sapiens chromosome 11 open reading frame 86 (C11orf86), mRNA [NM_001136485] |
| A_21_P0008828 | -3.7425687 |  |  |
| A_21_P0006564 | -3.7418432 | TSIX | Homo sapiens TSIX transcript, XIST antisense RNA (TSIX), antisense RNA [NR_003255] |
| A_21_P0003564 | -3.741118 |  |  |
| A_21_P0004114 | -3.7388072 |  | EST11177 human nasopharynx Homo sapiens cDNA, mRNA sequence [CD694654] |
| A_33_P3262083 | -3.7374272 | LOC100126784 | Homo sapiens uncharacterized LOC100126784 (LOC100126784), long non-coding RNA [NR_015384] |
| A_33_P3230668 | -3.7324982 | LOC100131232 | Homo sapiens cDNA FLJ33442 fis, clone BRACE2021936. [AK090761] |
| A_33_P3236628 | -3.7290046 | BCKDHA | Homo sapiens mRNA; cDNA DKFZp313C0640 (from clone DKFZp313C0640). [AL833143] |
| A_21_P0010167 | -3.7267113 |  |  |
| A_21_P0007839 | -3.7233746 |  |  |
| A_21_P0008138 | -3.7233303 |  |  |
| A_21_P0004917 | -3.722894 |  |  |
| A_21_P0002945 | -3.7177284 |  |  |
| A_24_P254705 | -3.7164385 | ZNF695 | Homo sapiens zinc finger protein 695 (ZNF695), transcript variant 1, mRNA [NM_020394] |
| A_33_P3227086 | -3.7154913 |  | Homo sapiens cDNA FLJ38343 fis, clone FCBBF3028472. [AK095662] |
| A_21_P0008129 | -3.7130017 |  |  |
| A_23_P161297 | -3.708394 | OGDHL | Homo sapiens oxoglutarate dehydrogenase-like (OGDHL), transcript variant 1, mRNA [NM_018245] |
| A_21_P0007333 | -3.7079165 |  |  |
| A_33_P3249259 | -3.706338 | TGM6 | Homo sapiens transglutaminase 6 (TGM6), transcript variant 1, mRNA [NM_198994] |
| A_19_P00808367 | -3.7060168 | PPP4R1L | Homo sapiens protein phosphatase 4, regulatory subunit 1-like (PPP4R1L), non-coding RNA [NR_003505] |
| A_24_P758256 | -3.701986 |  |  |
| A_21_P0000159 | -3.6970356 | ZNF568 | Homo sapiens zinc finger protein 568 (ZNF568), transcript variant 5, mRNA [NM_001204838] |
| A_33_P3344492 | -3.6903312 | SCARNA1 | Homo sapiens small Cajal body-specific RNA 1 (SCARNA1), guide RNA [NR_002997] |
| A_21_P0002775 | -3.68546 |  | BX116500 NCI_CGAP_Kid8 Homo sapiens cDNA clone IMAGp998A015526, mRNA sequence [BX116500] |
| A_24_P818010 | -3.6851203 | LOC729737 | Homo sapiens uncharacterized LOC729737 (LOC729737), long non-coding RNA [NR_039983] |
| A_21_P0013999 | -3.6814597 |  |  |
| A_21_P0005301 | -3.6809435 |  |  |
| A_33_P3248004 | -3.6797838 | FLJ41423 | Homo sapiens hypothetical LOC399886, mRNA (cDNA clone MGC:163508 IMAGE:40146667), complete cds. [BC130636] |
| A_33_P3229918 | -3.670446 | PTCRA | Homo sapiens pre T-cell antigen receptor alpha (PTCRA), transcript variant 1, mRNA [NM_001243168] |
| A_33_P3389779 | -3.6686618 |  | WD repeat domain 33 [Source:HGNC Symbol;Acc:25651] [ENST00000408998] |
| A_33_P3397127 | -3.6685033 |  | T cell receptor beta variable 6-2 (gene/pseudogene) [Source:HGNC Symbol;Acc:12227] [ENST00000562386] |
| A_33_P3383556 | -3.6681054 | SEC14L3 | Homo sapiens SEC14-like 3 (S. cerevisiae) (SEC14L3), transcript variant 1, mRNA [NM_174975] |
| A_21_P0005805 | -3.647332 |  |  |
| A_23_P82249 | -3.634063 | ABCB8 | Homo sapiens ATP-binding cassette, sub-family B (MDR/TAP), member 8 (ABCB8), transcript variant 2, mRNA [NM_007188] |
| A_21_P0000532 | -3.633985 | MGC27382 | Homo sapiens uncharacterized MGC27382 (MGC27382), long non-coding RNA [NR_027310] |
| A_23_P301846 | -3.6269536 | CALCA | Homo sapiens calcitonin-related polypeptide alpha (CALCA), transcript variant 2, mRNA [NM_001033952] |
| A_33_P3249305 | -3.6226544 | CHRD | Homo sapiens chordin (CHRD), mRNA [NM_003741] |
| A_33_P3329448 | -3.6165726 | MEIS1-AS3 | Homo sapiens MEIS1 antisense RNA 3 (MEIS1-AS3), long non-coding RNA [NR_046438] |
| A_33_P3268219 | -3.6132298 |  |  |
| A_23_P43800 | -3.611022 | BOP1 | Homo sapiens block of proliferation 1 (BOP1), mRNA [NM_015201] |
| A_33_P3263629 | -3.6081953 |  |  |
| A_23_P35316 | -3.6031108 | ZNF695 | Homo sapiens zinc finger protein 695 (ZNF695), transcript variant 1, mRNA [NM_020394] |
| A_21_P0011906 | -3.602775 | HS1BP3-IT1 | PREDICTED: Homo sapiens HS1BP3 intronic transcript 1 (non-protein coding) (HS1BP3-IT1), misc_RNA [XR_244594] |
| A_23_P4179 | -3.5967162 | LINC00483 | long intergenic non-protein coding RNA 483 [Source:HGNC Symbol;Acc:26080] [ENST00000300458] |
| A_33_P3313622 | -3.5939102 | MIR17HG | Homo sapiens miR-17-92 cluster host gene (non-protein coding) (MIR17HG), transcript variant 1, long non-coding RNA [NR_027350] |
| A_33_P3401284 | -3.5926287 | RMRP | Homo sapiens RNA component of mitochondrial RNA processing endoribonuclease (RMRP), RNase MRP RNA [NR_003051] |
| A_21_P0013569 | -3.5913649 | CNTNAP3 | Homo sapiens contactin associated protein-like 3 (CNTNAP3), mRNA [NM_033655] |
| A_21_P0004770 | -3.5784302 |  | AB371508 pGCAP10 [AB371508] |
| A_24_P13381 | -3.5764177 | TRPV4 | Homo sapiens transient receptor potential cation channel, subfamily V, member 4 (TRPV4), transcript variant 2, mRNA [NM_147204] |
| A_19_P00322673 | -3.57439 |  |  |
| A_21_P0005334 | -3.5739617 |  |  |
| A_23_P127079 | -3.5724466 | PPRC1 | Homo sapiens peroxisome proliferator-activated receptor gamma, coactivator-related 1 (PPRC1), mRNA [NM_015062] |
| A_21_P0010985 | -3.5626433 |  |  |
| A_33_P3281641 | -3.5625033 | OR4B1 | Homo sapiens olfactory receptor, family 4, subfamily B, member 1 (OR4B1), mRNA [NM_001005470] |
| A_19_P00319093 | -3.5591025 | LOC100506465 | Homo sapiens cDNA clone IMAGE:3836116, partial cds. [BC007399] |
| A_19_P00322173 | -3.556289 |  | UI-H-EU0-azk-g-10-0-UI.s1 NCI_CGAP_Car1 Homo sapiens cDNA clone IMAGE: 5849889 3', mRNA sequence [BQ182886] |
| A_21_P0011887 | -3.5540352 | LOC728763 | Putative ciliary rootlet coiled-coil protein-like 3 protein [Source:UniProtKB/Swiss-Prot;Acc:H7BZ55] [ENST00000430980] |
| A_21_P0003561 | -3.5535622 |  |  |
| A_33_P3389216 | -3.5350704 | DNM3OS | Homo sapiens DNM3 opposite strand/antisense RNA (DNM3OS), transcript variant 1, long non-coding RNA [NR_038397] |
| A_33_P3370832 | -3.5206568 | FAM117B | family with sequence similarity 117, member B [Source:HGNC Symbol;Acc:14440] [ENST00000481658] |
| A_21_P0000075 | -3.5115385 | SAP25 | Homo sapiens Sin3A-associated protein, 25kDa (SAP25), mRNA [NM_001168682] |
| A_21_P0006574 | -3.5093133 | LOC101928832 | PREDICTED: Homo sapiens uncharacterized LOC101928832 (LOC101928832), misc_RNA [XR_244520] |
| A_23_P35684 | -3.506275 | INPP5F | Homo sapiens inositol polyphosphate-5-phosphatase F (INPP5F), transcript variant 1, mRNA [NM_014937] |
| A_33_P3323847 | -3.5005894 | RECQL4 | Homo sapiens RecQ protein-like 4 (RECQL4), mRNA [NM_004260] |
| A_33_P3366141 | -3.4982278 | LOC100130269 | Homo sapiens cDNA FLJ36925 fis, clone BRACE2005169. [AK094244] |
| A_33_P3255070 | -3.497288 |  |  |
| A_33_P3290522 | -3.4961412 |  | AF076612 chordin {Homo sapiens} (exp=-1; wgp=0; cg=0), partial (3%) [THC2742735] |
| A_21_P0004175 | -3.488196 |  |  |
| A_23_P250156 | -3.4876304 | IGF2BP2 | Homo sapiens insulin-like growth factor 2 mRNA binding protein 2 (IGF2BP2), transcript variant 1, mRNA [NM_006548] |
| A_33_P3329326 | -3.4859545 | CD164L2 | CD164 sialomucin-like 2 [Source:HGNC Symbol;Acc:32043] [ENST00000374025] |
| A_33_P3288754 | -3.4839547 | C19orf48 | Homo sapiens chromosome 19 open reading frame 48 (C19orf48), mRNA [NM_199249] |
| A_33_P3227258 | -3.480024 |  |  |
| A_33_P3319134 | -3.4782786 | LOC100506191 | Homo sapiens clone pp9372 unknown mRNA. [AF289610] |
| A_21_P0004473 | -3.4764056 |  |  |
| A_21_P0000233 | -3.4717283 | SNORD38A | Homo sapiens small nucleolar RNA, C/D box 38A (SNORD38A), small nucleolar RNA [NR_001456] |
| A_21_P0008564 | -3.4683979 | HOMER2 | homer homolog 2 (Drosophila) [Source:HGNC Symbol;Acc:17513] [ENST00000500334] |
| A_32_P151800 | -3.454132 | FAM72D | Homo sapiens family with sequence similarity 72, member D (FAM72D), mRNA [NM_207418] |
| A_33_P3303016 | -3.4523845 |  | PREDICTED: Homo sapiens uncharacterized LOC100130587 (LOC100130587), misc_RNA [XR_248969] |
| A_24_P96780 | -3.4497347 | CENPF | Homo sapiens centromere protein F, 350/400kDa (CENPF), mRNA [NM_016343] |
| A_21_P0004107 | -3.4487894 |  |  |
| A_32_P504827 | -3.4352334 | MUC12 | Homo sapiens mucin 12, cell surface associated (MUC12), mRNA [NM_001164462] |
| A_23_P76102 | -3.4329739 | GDF11 | Homo sapiens growth differentiation factor 11 (GDF11), mRNA [NM_005811] |
| A_33_P3369446 | -3.4297976 |  |  |
| A_33_P3248962 | -3.4287694 | LOC100131742 | Homo sapiens cDNA FLJ26662 fis, clone MPG02040. [AK130172] |
| A_23_P134744 | -3.42343 | RNF122 | Homo sapiens ring finger protein 122 (RNF122), mRNA [NM_024787] |
| A_33_P3294886 | -3.4190094 | VPS52 | vacuolar protein sorting 52 homolog (S. cerevisiae) [Source:HGNC Symbol;Acc:10518] [ENST00000463641] |
| A_33_P3286916 | -3.4168835 | PDZD7 | Homo sapiens PDZ domain containing 7 (PDZD7), transcript variant 1, mRNA [NM_001195263] |
| A_33_P3274397 | -3.4098876 | CHM | Homo sapiens choroideremia (Rab escort protein 1) (CHM), transcript variant 2, mRNA [NM_001145414] |
| A_23_P142174 | -3.4056282 | FOXA3 | Homo sapiens forkhead box A3 (FOXA3), mRNA [NM_004497] |
| A_21_P0011838 | -3.399854 | ANAPC1 | Homo sapiens anaphase promoting complex subunit 1 (ANAPC1), mRNA [NM_022662] |
| A_33_P3386147 | -3.3987257 |  |  |
| A_21_P0014049 | -3.3915932 | LOC389834 | Homo sapiens ankyrin repeat domain 57 pseudogene (LOC389834), non-coding RNA [NR_027420] |
| A_21_P0012370 | -3.3911335 | LINC00693 | Homo sapiens long intergenic non-protein coding RNA 693 (LINC00693), long non-coding RNA [NR_038840] |
| A_24_P41939 | -3.390743 |  |  |
| A_33_P3261710 | -3.3828485 | FTLP10 | Homo sapiens ferritin, light polypeptide pseudogene 10 (FTLP10), non-coding RNA [NR_015446] |
| A_33_P3252839 | -3.3755143 | AZGP1P1 | Homo sapiens alpha-2-glycoprotein 1, zinc-binding pseudogene 1 (AZGP1P1), non-coding RNA [NR_036679] |
| A_21_P0013273 | -3.375477 |  |  |
| A_33_P3249897 | -3.3739903 |  |  |
| A_33_P3349469 | -3.3717935 | ATAD3A | Homo sapiens ATPase family, AAA domain containing 3A (ATAD3A), transcript variant 1, mRNA [NM_018188] |
| A_32_P213637 | -3.3689475 | LINC01061 | Homo sapiens long intergenic non-protein coding RNA 1061 (LINC01061), long non-coding RNA [NR_037596] |
| A_21_P0009803 | -3.368404 |  |  |
| A_33_P3421748 | -3.363564 |  | AF380183 SON DNA binding protein isoform E {Homo sapiens} (exp=-1; wgp=0; cg=0), partial (7%) [THC2515264] |
| A_21_P0000215 | -3.3618963 | SNORD55 | Homo sapiens small nucleolar RNA, C/D box 55 (SNORD55), small nucleolar RNA [NR_000015] |
| A_21_P0011954 | -3.356974 | LOC101929886 | PREDICTED: Homo sapiens uncharacterized LOC101929886 (LOC101929886), transcript variant X1, misc_RNA [XR_251154] |
| A_21_P0002782 | -3.3522747 |  | DA149036 BRAMY2 Homo sapiens cDNA clone BRAMY2006448 5', mRNA sequence [DA149036] |
| A_33_P3807062 | -3.3522036 | HJURP | Homo sapiens Holliday junction recognition protein (HJURP), transcript variant 1, mRNA [NM_018410] |
| A_33_P3312251 | -3.3502774 |  |  |
| A_33_P3412335 | -3.347658 | LOC100128356 | Homo sapiens protein transactivated by hepatitis B virus E antigen mRNA, complete cds. [AY423624] |
| A_21_P0000301 | -3.3448408 | SNORA5A | Homo sapiens small nucleolar RNA, H/ACA box 5A (SNORA5A), small nucleolar RNA [NR_002919] |
| A_33_P3334448 | -3.3432765 | SNORA62 | Homo sapiens small nucleolar RNA, H/ACA box 62 (SNORA62), small nucleolar RNA [NR_002324] |
| A_21_P0007623 | -3.3423257 |  |  |
| A_21_P0011005 | -3.3419292 |  |  |
| A_33_P3291816 | -3.3409386 | CLDN22 | Homo sapiens claudin 22 (CLDN22), mRNA [NM_001111319] |
| A_23_P131588 | -3.3398657 | BMP10 | Homo sapiens bone morphogenetic protein 10 (BMP10), mRNA [NM_014482] |
| A_21_P0001374 | -3.3394823 | NBPF15 | neuroblastoma breakpoint family, member 15 [Source:HGNC Symbol;Acc:28791] [ENST00000464336] |
| A_23_P52017 | -3.3372731 | ASPM | Homo sapiens asp (abnormal spindle) homolog, microcephaly associated (Drosophila) (ASPM), transcript variant 1, mRNA [NM_018136] |
| A_23_P163209 | -3.336556 | BCL2L10 | Homo sapiens BCL2-like 10 (apoptosis facilitator) (BCL2L10), mRNA [NM_020396] |
| A_33_P3855484 | -3.3365345 |  |  |
| A_33_P3239298 | -3.3321323 | PRODH | proline dehydrogenase (oxidase) 1 [Source:HGNC Symbol;Acc:9453] [ENST00000399694] |
| ERCC-00086_228 | -3.331335 |  |  |
| A_24_P931736 | -3.331215 | LOC100133106 | Homo sapiens clone DNA175753 VCEW9374 (UNQ9374) mRNA, complete cds. [AY358216] |
| A_33_P3263523 | -3.3261046 | C1orf35 | Homo sapiens chromosome 1 open reading frame 35 (C1orf35), mRNA [NM_024319] |
| A_21_P0002562 | -3.3141341 |  |  |
| A_21_P0004629 | -3.3128126 |  |  |
| A_23_P204364 | -3.3085399 | NOP2 | Homo sapiens NOP2 nucleolar protein (NOP2), transcript variant 2, mRNA [NM_001033714] |
| A_23_P115482 | -3.3039298 | UBE2T | Homo sapiens ubiquitin-conjugating enzyme E2T (putative) (UBE2T), mRNA [NM_014176] |
| A_32_P180741 | -3.30328 | TNK2 | Homo sapiens tyrosine kinase, non-receptor, 2 (TNK2), transcript variant 2, mRNA [NM_001010938] |
| A_33_P3223663 | -3.2982306 |  |  |
| A_21_P0012112 | -3.2926502 |  |  |
| A_21_P0000463 | -3.2926338 | SNORD105B | Homo sapiens small nucleolar RNA, C/D box 105B (SNORD105B), small nucleolar RNA [NR_003688] |
| A_33_P3288159 | -3.2923517 | ASPM | Homo sapiens asp (abnormal spindle) homolog, microcephaly associated (Drosophila) (ASPM), transcript variant 1, mRNA [NM_018136] |
| A_21_P0011674 | -3.2911983 |  |  |
| A_23_P98898 | -3.2880979 | CDK2 | Homo sapiens cyclin-dependent kinase 2 (CDK2), transcript variant 1, mRNA [NM_001798] |
| A_21_P0006308 | -3.276289 |  |  |
| A_33_P3248028 | -3.276128 |  |  |
| A_33_P3229552 | -3.2714427 | LRFN1 | Homo sapiens leucine rich repeat and fibronectin type III domain containing 1 (LRFN1), mRNA [NM_020862] |
| A_33_P3262138 | -3.2691612 | ANAPC1 | Homo sapiens anaphase promoting complex subunit 1 (ANAPC1), mRNA [NM_022662] |
| A_21_P0006732 | -3.2674835 |  | XM_750535 peroxisomal membrane anchor protein {Aspergillus fumigatus Af293} (exp=-1; wgp=0; cg=0), partial (4%) [THC2741636] |
| A_33_P3274731 | -3.2565985 | CLTC | Homo sapiens clathrin, heavy chain (Hc) (CLTC), mRNA [NM_004859] |
| A_21_P0014828 | -3.2511892 |  |  |
| A_21_P0000314 | -3.2440143 | SNORA17 | Homo sapiens small nucleolar RNA, H/ACA box 17 (SNORA17), small nucleolar RNA [NR_002958] |
| A_33_P3351440 | -3.2417238 |  | BC039396 FAM19A5 protein {Homo sapiens} (exp=-1; wgp=0; cg=0), partial (39%) [THC2731763] |
| A_21_P0014029 | -3.2414737 |  | scaffolding protein involved in DNA repair [Source:HGNC Symbol;Acc:28971] [ENST00000521798] |
| A_33_P3414594 | -3.2208586 | NR1I3 | Homo sapiens nuclear receptor subfamily 1, group I, member 3 (NR1I3), transcript variant 8, mRNA [NM_001077474] |
| A_24_P55148 | -3.220225 | HIST1H2BJ | Homo sapiens histone cluster 1, H2bj (HIST1H2BJ), mRNA [NM_021058] |
| A_32_P11230 | -3.219944 | LINC00999 | Homo sapiens long intergenic non-protein coding RNA 999 (LINC00999), long non-coding RNA [NR_024497] |
| A_33_P3390673 | -3.2193184 |  |  |
| A_21_P0000850 | -3.2160273 | LOC100506343 | Homo sapiens uncharacterized LOC100506343 (LOC100506343), transcript variant 1, long non-coding RNA [NR_038951] |
| A_33_P3221055 | -3.2147973 | LOC100996442 | PREDICTED: Homo sapiens uncharacterized LOC100996442 (LOC100996442), transcript variant X1, misc_RNA [XR_159064] |
| A_21_P0009741 | -3.212141 |  |  |
| A_33_P3258223 | -3.2113206 | MCM7 | Homo sapiens minichromosome maintenance complex component 7 (MCM7), transcript variant 1, mRNA [NM_005916] |
| A_21_P0010922 | -3.2089467 | BEND3P3 | Homo sapiens BEN domain containing 3 pseudogene 3 (BEND3P3), non-coding RNA [NR_027512] |
| A_21_P0010672 | -3.2075093 |  |  |
| A_33_P3313552 | -3.2069385 |  |  |
| A_21_P0014243 | -3.1996586 |  | Q8NE53_HUMAN (Q8NE53) Zinedin, partial (3%) [THC2628554] |
| A_23_P377212 | -3.198652 | MYEOV2 | Homo sapiens myeloma overexpressed 2 (MYEOV2), transcript variant 1, mRNA [NM_138336] |
| A_21_P0002706 | -3.1931186 |  | Homo sapiens cDNA FLJ25897 fis, clone CBR03768. [AK098763] |
| A_24_P208081 | -3.1822338 | PLA2G2F | Homo sapiens phospholipase A2, group IIF (PLA2G2F), mRNA [NM_022819] |
| A_33_P3257252 | -3.1799335 | ZNF551 | Homo sapiens zinc finger protein 551 (ZNF551), transcript variant 1, mRNA [NM_138347] |
| A_21_P0000234 | -3.1784093 | SNORD38B | Homo sapiens small nucleolar RNA, C/D box 38B (SNORD38B), small nucleolar RNA [NR_001457] |
| A_24_P223384 | -3.1778505 | HIST1H2AB | Homo sapiens histone cluster 1, H2ab (HIST1H2AB), mRNA [NM_003513] |
| A_21_P0003385 | -3.171858 |  |  |
| A_21_P0007651 | -3.1704602 |  |  |
| A_33_P3247799 | -3.1646898 |  |  |
| A_24_P943106 | -3.1638823 | U2SURP | Homo sapiens U2 snRNP-associated SURP domain containing (U2SURP), mRNA [NM_001080415] |
| A_33_P3286709 | -3.1627376 |  |  |
| A_23_P129312 | -3.1617973 | PPP1R14D | Homo sapiens protein phosphatase 1, regulatory (inhibitor) subunit 14D (PPP1R14D), transcript variant 1, mRNA [NM_017726] |
| A_33_P3332348 | -3.1611934 | RN7SL1 | Homo sapiens RNA, 7SL, cytoplasmic 1 (RN7SL1), SRP RNA [NR_002715] |
| A_21_P0013705 | -3.1571877 |  |  |
| A_21_P0009004 | -3.1523142 |  |  |
| A_33_P3239222 | -3.1519544 | EIF3B | Homo sapiens eukaryotic translation initiation factor 3, subunit B (EIF3B), transcript variant 2, mRNA [NM_001037283] |
| A_19_P00321983 | -3.1477947 |  | Homo sapiens cDNA, FLJ99758. [AK309717] |
| A_21_P0008836 | -3.1472979 |  | Synthetic construct Homo sapiens gateway clone IMAGE:100017125 3' read UBE2Q2 mRNA. [CU676033] |
| A_23_P363954 | -3.147211 | THRSP | Homo sapiens thyroid hormone responsive (THRSP), mRNA [NM_003251] |
| A_33_P3234303 | -3.1454146 | PAX6 | paired box 6 [Source:HGNC Symbol;Acc:8620] [ENST00000379123] |
| A_21_P0002009 | -3.1439497 | LINC00298 | Homo sapiens long intergenic non-protein coding RNA 298 (LINC00298), long non-coding RNA [NR_015405] |
| A_32_P141238 | -3.1436658 | ANO2 | Homo sapiens anoctamin 2 (ANO2), transcript variant 2, mRNA [NM_001278597] |
| A_21_P0012859 | -3.1432958 | LOC255187 |  |
| A_21_P0008479 | -3.1428883 |  |  |
| A_21_P0014595 | -3.1400788 |  | PREDICTED: Homo sapiens uncharacterized LOC101927472 (LOC101927472), misc_RNA [XR_246182] |
| A_33_P3375636 | -3.139715 | FAM196B | Homo sapiens family with sequence similarity 196, member B (FAM196B), mRNA [NM_001129891] |
| A_23_P164826 | -3.137601 | RNASEH2A | Homo sapiens ribonuclease H2, subunit A (RNASEH2A), mRNA [NM_006397] |
| A_21_P0000560 | -3.131471 | TMEM30C | Homo sapiens transmembrane protein 30C (TMEM30C), transcript variant 2, non-coding RNA [NR_028358] |
| A_24_P298027 | -3.1249118 | AXIN2 | Homo sapiens axin 2 (AXIN2), mRNA [NM_004655] |
| A_33_P3264193 | -3.1230006 | OR2D2 | Homo sapiens olfactory receptor, family 2, subfamily D, member 2 (OR2D2), mRNA [NM_003700] |
| A_33_P3279851 | -3.1225147 | ANAPC1P1 | Homo sapiens anaphase promoting complex subunit 1 pseudogene 1 (ANAPC1P1), non-coding RNA [NR_037931] |
| ERCC-00014_76 | -3.121997 |  |  |
| A_21_P0010347 | -3.1188643 | MIAT | myocardial infarction associated transcript (non-protein coding) [Source:HGNC Symbol;Acc:33425] [ENST00000430483] |
| A_33_P3266609 | -3.117893 |  |  |
| A_23_P405161 | -3.1168966 | C7orf33 | Homo sapiens chromosome 7 open reading frame 33 (C7orf33), mRNA [NM_145304] |
| A_24_P652502 | -3.1157126 |  | T cell receptor alpha variable 4 [Source:HGNC Symbol;Acc:12140] [ENST00000390426] |
| A_24_P34545 | -3.114601 | ING5 | Homo sapiens inhibitor of growth family, member 5 (ING5), mRNA [NM_032329] |
| A_21_P0014819 | -3.1143231 |  |  |
| A_33_P3332871 | -3.1118054 |  | 601279876F1 NIH_MGC_39 Homo sapiens cDNA clone IMAGE:3621930 5', mRNA sequence [BE616696] |
| A_23_P37718 | -3.1108973 | CNGB1 | Homo sapiens cyclic nucleotide gated channel beta 1 (CNGB1), transcript variant 1, mRNA [NM_001297] |
| A_21_P0006423 | -3.1099114 |  | imageqc_2_2001 [BM083567] |
| A_33_P3322724 | -3.1058109 | POLR3A | Homo sapiens polymerase (RNA) III (DNA directed) polypeptide A, 155kDa (POLR3A), mRNA [NM_007055] |
| A_23_P346405 | -3.103626 | MCU | Homo sapiens mitochondrial calcium uniporter (MCU), transcript variant 1, mRNA [NM_138357] |
| A_21_P0007646 | -3.102018 |  |  |
| A_33_P3332248 | -3.1006522 | ADRA1A | adrenoceptor alpha 1A [Source:HGNC Symbol;Acc:277] [ENST00000380573] |
| A_33_P3217020 | -3.0981486 | RAPH1 | Homo sapiens Ras association (RalGDS/AF-6) and pleckstrin homology domains 1 (RAPH1), transcript variant 3, mRNA [NM_203365] |
| A_21_P0006941 | -3.095466 |  |  |
| A_33_P3295786 | -3.094443 | FAM159A | Homo sapiens family with sequence similarity 159, member A (FAM159A), mRNA [NM_001042693] |
| A_21_P0001387 | -3.094412 |  | PREDICTED: Homo sapiens uncharacterized LOC101930532 (LOC101930532), misc_RNA [XR_248664] |
| A_33_P3305325 | -3.0925896 |  |  |
| A_33_P3317825 | -3.0917828 | NRAS | Homo sapiens neuroblastoma RAS viral (v-ras) oncogene homolog (NRAS), mRNA [NM_002524] |
| A_33_P3249037 | -3.0866451 | PRRG3 | proline rich Gla (G-carboxyglutamic acid) 3 (transmembrane) [Source:HGNC Symbol;Acc:30798] [ENST00000370354] |
| A_33_P3310274 | -3.0845041 |  | Q5T511_HUMAN (Q5T511) Leucine-rich repeat-containing G protein-coupled receptor 6 (Fragment), complete [THC2755556] |
| A_21_P0005475 | -3.084375 |  | BX100853 NCI_CGAP_Pr28 Homo sapiens cDNA clone IMAGp998E195740, mRNA sequence [BX100853] |
| A_33_P3358469 | -3.0831363 | GLI2 | Homo sapiens GLI family zinc finger 2 (GLI2), mRNA [NM_005270] |
| A_21_P0011557 | -3.0798485 |  |  |
| A_33_P3407618 | -3.0795908 | LOC727751 | Homo sapiens golgin A2 pseudogene (LOC727751), transcript variant 1, non-coding RNA [NR_102747] |
| A_23_P137470 | -3.0790515 | SIPA1L2 | Homo sapiens signal-induced proliferation-associated 1 like 2 (SIPA1L2), mRNA [NM_020808] |
| A_21_P0003465 | -3.0758278 |  |  |
| A_21_P0008026 | -3.0747216 |  |  |
| A_21_P0007171 | -3.0742958 |  |  |
| A_33_P3334436 | -3.0708077 |  |  |
| A_33_P3382758 | -3.0635707 |  | BX404796 Homo sapiens FETAL LIVER Homo sapiens cDNA clone CS0DM011YO11 5-PRIME, mRNA sequence [BX404796] |
| A_33_P3372705 | -3.0534122 | SRGAP2B | Homo sapiens SLIT-ROBO Rho GTPase activating protein 2B (SRGAP2B), mRNA [NM_001271870] |
| A_23_P73982 | -3.0505238 | NDC1 | Homo sapiens NDC1 transmembrane nucleoporin (NDC1), transcript variant 1, mRNA [NM_018087] |
| A_21_P0002720 | -3.0490108 |  |  |
| A_33_P3213526 | -3.048921 |  |  |
| A_33_P3251198 | -3.048914 | PLCB1 | phospholipase C, beta 1 (phosphoinositide-specific) [Source:HGNC Symbol;Acc:15917] [ENST00000404098] |
| A_33_P3228445 | -3.0475433 | FXYD2 | Homo sapiens FXYD domain containing ion transport regulator 2 (FXYD2), transcript variant a, mRNA [NM_001680] |
| A_23_P157072 | -3.046437 | EIF3B | Homo sapiens eukaryotic translation initiation factor 3, subunit B (EIF3B), transcript variant 2, mRNA [NM_001037283] |
| A_21_P0014507 | -3.04534 |  |  |
| A_33_P3379001 | -3.0451896 | UNC5A | Homo sapiens unc-5 homolog A (C. elegans) (UNC5A), mRNA [NM_133369] |
| A_24_P158468 | -3.0417218 | C10orf91 | Homo sapiens chromosome 10 open reading frame 91 (C10orf91), mRNA [NM_173541] |
| A_23_P64825 | -3.041021 | LACRT | Homo sapiens lacritin (LACRT), mRNA [NM_033277] |
| A_21_P0011500 | -3.0384667 | LOC100288069 | Homo sapiens uncharacterized LOC100288069 (LOC100288069), long non-coding RNA [NR_033908] |
| A_24_P376441 | -3.0355742 | TAF5L | Homo sapiens TAF5-like RNA polymerase II, p300/CBP-associated factor (PCAF)-associated factor, 65kDa (TAF5L), transcript variant 2, mRNA [NM_001025247] |
| A_33_P3311618 | -3.0354292 | RABGGTB | Homo sapiens Rab geranylgeranyltransferase, beta subunit (RABGGTB), transcript variant 1, mRNA [NM_004582] |
| A_33_P3420611 | -3.0327163 | OR4D2 | Homo sapiens olfactory receptor, family 4, subfamily D, member 2 (OR4D2), mRNA [NM_001004707] |
| A_21_P0009717 | -3.0322728 |  |  |
| A_33_P3238325 | -3.0304706 |  |  |
| A_19_P00321178 | -3.02934 |  |  |
| A_33_P3253179 | -3.0286822 |  |  |
| A_33_P3279362 | -3.0284715 | GRIK1-AS1 | Homo sapiens GRIK1 antisense RNA 1 (GRIK1-AS1), long non-coding RNA [NR_027021] |
| A_33_P3421275 | -3.0262508 | NKX6-3 | Homo sapiens NK6 homeobox 3 (NKX6-3), mRNA [NM_152568] |
| A_23_P106236 | -3.0253754 | FOXN3-AS2 | Homo sapiens FOXN3 antisense RNA 2 (FOXN3-AS2), long non-coding RNA [NR_024620] |
| A_33_P3423740 | -3.0242493 | TMEM164 | Homo sapiens transmembrane protein 164 (TMEM164), transcript variant 2, mRNA [NM_032227] |
| A_21_P0004150 | -3.0237825 |  | RST42639 Athersys RAGE Library Homo sapiens cDNA, mRNA sequence [BG460179] |
| A_21_P0000392 | -3.022847 | SNORD98 | Homo sapiens small nucleolar RNA, C/D box 98 (SNORD98), small nucleolar RNA [NR_003076] |
| A_33_P3312044 | -3.0221596 |  |  |
| A_33_P3398000 | -3.0186887 | TTN-AS1 | Homo sapiens TTN antisense RNA 1 (TTN-AS1), transcript variant 1, long non-coding RNA [NR_038272] |
| A_33_P3253501 | -3.015294 | HIST2H2BF | Homo sapiens histone cluster 2, H2bf (HIST2H2BF), transcript variant 1, mRNA [NM_001024599] |
| A_33_P3311210 | -3.0148263 | BSX | Homo sapiens brain-specific homeobox (BSX), mRNA [NM_001098169] |
| A_33_P3312366 | -3.011916 | TOP3B | Homo sapiens topoisomerase (DNA) III beta (TOP3B), transcript variant 1, mRNA [NM_003935] |
| A_21_P0002042 | -3.0063255 |  | DB066812 TESTI4 Homo sapiens cDNA clone TESTI4008254 5', mRNA sequence [DB066812] |
| A_23_P202143 | -2.999731 | NOLC1 | Homo sapiens nucleolar and coiled-body phosphoprotein 1 (NOLC1), transcript variant 1, mRNA [NM_001284388] |
| A_19_P00809382 | -2.9989135 | SETD1B | Homo sapiens SET domain containing 1B (SETD1B), mRNA [NM_015048] |
| A_23_P431381 | -2.9985528 | C14orf80 | Homo sapiens chromosome 14 open reading frame 80 (C14orf80), transcript variant 1, mRNA [NM_001134875] |
| A_33_P3402225 | -2.9970798 |  |  |
| A_33_P3314352 | -2.9967942 |  |  |
| A_21_P0010568 | -2.9957306 |  |  |
| A_33_P3244895 | -2.9904194 | TMCC2 | Homo sapiens clone DNA142934 GLSH6409 (UNQ6409) mRNA, complete cds. [AY358192] |
| A_33_P3229067 | -2.9902592 | HIST1H2BN | Homo sapiens histone cluster 1, H2bn (HIST1H2BN), mRNA [NM_003520] |
| A_23_P51410 | -2.9870825 | SMYD3 | Homo sapiens SET and MYND domain containing 3 (SMYD3), transcript variant 2, mRNA [NM_022743] |
| A_33_P3393456 | -2.9849324 | LOC100131129 | Homo sapiens cDNA FLJ45249 fis, clone BRHIP2006921. [AK127184] |
| A_33_P3284077 | -2.9837527 | NUP133 | Homo sapiens nucleoporin 133kDa (NUP133), mRNA [NM_018230] |
| A_33_P3409184 | -2.9824107 |  |  |
| A_23_P156310 | -2.9816568 | SKP2 | Homo sapiens S-phase kinase-associated protein 2, E3 ubiquitin protein ligase (SKP2), transcript variant 2, mRNA [NM_032637] |
| A_23_P23616 | -2.98053 | PLEKHN1 | Homo sapiens pleckstrin homology domain containing, family N member 1 (PLEKHN1), transcript variant 1, mRNA [NM_032129] |
| A_23_P16683 | -2.9759738 | TRMT1 | Homo sapiens tRNA methyltransferase 1 homolog (S. cerevisiae) (TRMT1), transcript variant 1, mRNA [NM_017722] |
| A_21_P0007134 | -2.9703183 | LINC00678 | Homo sapiens long intergenic non-protein coding RNA 678 (LINC00678), long non-coding RNA [NR_102708] |
| A_21_P0000294 | -2.9685178 | SNORA71B | Homo sapiens small nucleolar RNA, H/ACA box 71B (SNORA71B), small nucleolar RNA [NR_002910] |
| A_33_P3216357 | -2.968477 | KDM5C | Homo sapiens lysine (K)-specific demethylase 5C (KDM5C), transcript variant 2, mRNA [NM_001146702] |
| A_33_P3388482 | -2.9667869 | TMEM120B | Homo sapiens transmembrane protein 120B (TMEM120B), mRNA [NM_001080825] |
| A_33_P3363465 | -2.9660628 |  | Homo sapiens cDNA FLJ43298 fis, clone NCRRP1000129. [AK125288] |
| A_21_P0004013 | -2.9654214 |  |  |
| A_21_P0000212 | -2.9629304 | SNORA68 | Homo sapiens small nucleolar RNA, H/ACA box 68 (SNORA68), small nucleolar RNA [NR_000012] |
| ERCC-00081_60 | -2.961595 |  |  |
| A_21_P0011009 | -2.9589493 |  |  |
| A_23_P11256 | -2.9568667 | MAGEE2 | Homo sapiens melanoma antigen family E, 2 (MAGEE2), mRNA [NM_138703] |
| A_21_P0013479 | -2.9564166 |  |  |
| A_21_P0001976 | -2.9539583 | LOC151484 |  |
| A_33_P3266192 | -2.9538794 | GAS6-AS1 | Homo sapiens hypothetical LOC650669, mRNA (cDNA clone MGC:169000 IMAGE:9021377), complete cds. [BC137379] |
| A_21_P0009835 | -2.953028 | LOC100270679 | Homo sapiens uncharacterized LOC100270679 (LOC100270679), long non-coding RNA [NR_038394] |
| A_33_P3291796 | -2.9515593 | CAMKV | Homo sapiens CaM kinase-like vesicle-associated (CAMKV), mRNA [NM_024046] |
| A_23_P435941 | -2.951042 | SAMD1 | Homo sapiens sterile alpha motif domain containing 1 (SAMD1), mRNA [NM_138352] |
| A_33_P3215487 | -2.9496057 | AP5Z1 | Homo sapiens adaptor-related protein complex 5, zeta 1 subunit (AP5Z1), mRNA [NM_014855] |
| A_24_P363477 | -2.946887 | FTSJ2 | Homo sapiens FtsJ RNA methyltransferase homolog 2 (E. coli) (FTSJ2), mRNA [NM_013393] |
| A_33_P3317073 | -2.944728 | MOB1B | Homo sapiens MOB kinase activator 1B (MOB1B), transcript variant 1, mRNA [NM_001244766] |
| A_33_P3301524 | -2.940574 | XRCC3 | Homo sapiens X-ray repair complementing defective repair in Chinese hamster cells 3 (XRCC3), transcript variant 1, mRNA [NM_001100119] |
| A_33_P3245947 | -2.9350796 | LRRC45 | Homo sapiens leucine rich repeat containing 45 (LRRC45), mRNA [NM_144999] |
| A_33_P3256858 | -2.932347 | C14orf80 | Homo sapiens chromosome 14 open reading frame 80 (C14orf80), transcript variant 1, mRNA [NM_001134875] |
| A_33_P3255677 | -2.930714 |  |  |
| A_21_P0012519 | -2.9297454 |  | DB231946 TRACH3 Homo sapiens cDNA clone TRACH3025715 5', mRNA sequence [DB231946] |
| A_21_P0008964 | -2.929528 |  |  |
| A_21_P0006476 | -2.929055 |  |  |
| A_21_P0009973 | -2.921964 |  |  |
| A_21_P0009992 | -2.9202726 |  |  |
| A_23_P4133 | -2.9160008 | AOC2 | Homo sapiens amine oxidase, copper containing 2 (retina-specific) (AOC2), transcript variant 1, mRNA [NM_001158] |
| A_33_P3354553 | -2.915941 |  |  |
| A_23_P28538 | -2.9135094 | MRPS5 | Homo sapiens mitochondrial ribosomal protein S5 (MRPS5), mRNA [NM_031902] |
| A_21_P0006948 | -2.912854 |  |  |
| A_32_P75425 | -2.9105043 | LINC01061 | Homo sapiens long intergenic non-protein coding RNA 1061 (LINC01061), long non-coding RNA [NR_037596] |
| A_33_P3239579 | -2.9080844 | LOC100131060 | Homo sapiens uncharacterized LOC100131060 (LOC100131060), long non-coding RNA [NR_034014] |
| A_21_P0001696 | -2.907054 | LOC101928334 | PREDICTED: Homo sapiens uncharacterized LOC101928334 (LOC101928334), transcript variant X1, misc_RNA [XR_246382] |
| A_33_P3529859 | -2.9006865 | KDM2B | Homo sapiens lysine (K)-specific demethylase 2B (KDM2B), transcript variant 1, mRNA [NM_032590] |
| A_23_P110851 | -2.9003544 | TERT | Homo sapiens telomerase reverse transcriptase (TERT), transcript variant 1, mRNA [NM_198253] |
| A_21_P0011833 | -2.8980775 | LINC01127 | Homo sapiens long intergenic non-protein coding RNA 1127 (LINC01127), long non-coding RNA [NR_103791] |
| A_21_P0006021 | -2.888067 |  |  |
| A_33_P3722782 | -2.8873124 | TMEM72-AS1 | Homo sapiens TMEM72 antisense RNA 1 (TMEM72-AS1), long non-coding RNA [NR_033842] |
| A_21_P0012123 | -2.8870552 | LOC100505663 | PREDICTED: Homo sapiens uncharacterized LOC100505663 (LOC100505663), transcript variant X2, misc_RNA [XR_109600] |
| A_21_P0000275 | -2.8856199 | SNORD96A | Homo sapiens small nucleolar RNA, C/D box 96A (SNORD96A), small nucleolar RNA [NR_002592] |
| A_21_P0000288 | -2.8851116 | SNORD45B | Homo sapiens small nucleolar RNA, C/D box 45B (SNORD45B), small nucleolar RNA [NR_002748] |
| A_23_P318296 | -2.8823705 | PLEKHA8 | Homo sapiens pleckstrin homology domain containing, family A (phosphoinositide binding specific) member 8 (PLEKHA8), transcript variant 3, mRNA [NM_032639] |
| A_21_P0011429 | -2.8789196 | HERC2 | Homo sapiens HECT and RLD domain containing E3 ubiquitin protein ligase 2 (HERC2), mRNA [NM_004667] |
| A_33_P3317442 | -2.878336 | MCF2L | Homo sapiens MCF.2 cell line derived transforming sequence-like (MCF2L), transcript variant 2, mRNA [NM_024979] |
| A_33_P3216621 | -2.8778849 |  |  |
| A_33_P3424516 | -2.866065 | OR51M1 | Homo sapiens olfactory receptor, family 51, subfamily M, member 1 (OR51M1), mRNA [NM_001004756] |
| A_21_P0012012 | -2.8643084 |  | Q5QPL3_HUMAN (Q5QPL3) Novel zinc finger protein (Fragment), partial (15%) [THC2615552] |
| A_21_P0000343 | -2.8628023 | SNORA5C | Homo sapiens small nucleolar RNA, H/ACA box 5C (SNORA5C), small nucleolar RNA [NR_002991] |
| A_21_P0005226 | -2.8616521 |  |  |
| A_24_P179013 | -2.854871 |  | GB |
| A_21_P0012877 | -2.8546784 | LOC100288069 | Homo sapiens uncharacterized LOC100288069 (LOC100288069), long non-coding RNA [NR_033908] |
| A_33_P3392097 | -2.8541634 |  |  |
| A_23_P115838 | -2.850793 | C10orf12 | Homo sapiens chromosome 10 open reading frame 12 (C10orf12), mRNA [NM_015652] |
| A_21_P0010512 | -2.8501866 | LOC728716 | Homo sapiens uncharacterized LOC728716 (LOC728716), long non-coding RNA [NR_040065] |
| A_33_P3272330 | -2.8454797 | DNMT3A | Homo sapiens DNA (cytosine-5-)-methyltransferase 3 alpha (DNMT3A), transcript variant 1, mRNA [NM_175629] |
| A_33_P3220386 | -2.844775 | LOC157273 | Homo sapiens LP5624 mRNA, complete cds. [AY203962] |
| A_33_P3465703 | -2.8405843 | SNORA60 | 601888042F1 NIH_MGC_17 Homo sapiens cDNA clone IMAGE:4121819 5', mRNA sequence [BF304636] |
| A_23_P171258 | -2.839147 | ABCB7 | Homo sapiens ATP-binding cassette, sub-family B (MDR/TAP), member 7 (ABCB7), transcript variant 1, mRNA [NM_004299] |
| A_21_P0013570 | -2.8380017 |  |  |
| A_21_P0013608 | -2.8374102 | LOC101928483 | PREDICTED: Homo sapiens uncharacterized LOC101928483 (LOC101928483), transcript variant X2, misc_RNA [XR_252671] |
| A_33_P3344603 | -2.8347843 | ZNF280D | Homo sapiens zinc finger protein 280D (ZNF280D), transcript variant 3, mRNA [NM_001002844] |
| A_21_P0001230 | -2.832367 | LOC101927143 | PREDICTED: Homo sapiens uncharacterized LOC101927143 (LOC101927143), misc_RNA [XR_247080] |
| A_33_P3210079 | -2.8236015 | SF3A2 | Homo sapiens splicing factor 3a, subunit 2, 66kDa (SF3A2), mRNA [NM_007165] |
| A_21_P0011467 | -2.814784 | HERC2 | Homo sapiens HECT and RLD domain containing E3 ubiquitin protein ligase 2 (HERC2), mRNA [NM_004667] |
| A_21_P0014809 | -2.8103912 | FAM136A | Homo sapiens family with sequence similarity 136, member A (FAM136A), mRNA [NM_032822] |
| A_23_P98645 | -2.8069222 | DCHS1 | Homo sapiens dachsous cadherin-related 1 (DCHS1), mRNA [NM_003737] |
| A_21_P0011299 | -2.8066056 |  |  |
| A_21_P0002196 | -2.8029127 | LINC00486 | PREDICTED: Homo sapiens long intergenic non-protein coding RNA 486 (LINC00486), transcript variant X1, misc_RNA [XR_244929] |
| A_21_P0008760 | -2.7997336 |  |  |
| A_33_P3273752 | -2.797051 |  |  |
| A_33_P3264179 | -2.7964501 | LCE3E | Homo sapiens late cornified envelope 3E (LCE3E), mRNA [NM_178435] |
| A_33_P3400152 | -2.795011 | ENTPD1-AS1 | Homo sapiens ENTPD1 antisense RNA 1 (ENTPD1-AS1), long non-coding RNA [NR_038444] |
| A_23_P111112 | -2.7933776 | VARS2 | Homo sapiens valyl-tRNA synthetase 2, mitochondrial (VARS2), transcript variant 2, mRNA [NM_020442] |
| A_21_P0001095 | -2.7920725 |  | AGENCOURT_7905504 NIH_MGC_82 Homo sapiens cDNA clone IMAGE:6105558 5', mRNA sequence [BQ435581] |
| A_23_P50928 | -2.7913897 | C1QL2 | Homo sapiens complement component 1, q subcomponent-like 2 (C1QL2), mRNA [NM_182528] |
| A_33_P3327567 | -2.7913594 | TMEM41A | transmembrane protein 41A [Source:HGNC Symbol;Acc:30544] [ENST00000467061] |
| A_21_P0013930 | -2.7861238 | LOC100287852 | PREDICTED: Homo sapiens protein FAM136A-like (LOC100287852), mRNA [XM_002342390] |
| A_33_P3218559 | -2.785048 |  |  |
| A_21_P0001542 | -2.7846756 |  |  |
| A_33_P3410507 | -2.7842643 | CEP170 | Homo sapiens centrosomal protein 170kDa (CEP170), transcript variant alpha, mRNA [NM_014812] |
| A_23_P44996 | -2.7816634 | S100G | Homo sapiens S100 calcium binding protein G (S100G), mRNA [NM_004057] |
| A_33_P3407157 | -2.7814763 |  | DB050195 TESTI2 Homo sapiens cDNA clone TESTI2039685 5', mRNA sequence [DB050195] |
| ERCC-00058_185 | -2.7785466 |  |  |
| (+)E1A_r60_a107 | -2.77551 |  | Unknown |
| A_32_P232192 | -2.7733169 | DIS3L2 | Homo sapiens DIS3 mitotic control homolog (S. cerevisiae)-like 2 (DIS3L2), transcript variant 2, mRNA [NM_001257281] |
| A_33_P3325229 | -2.7722342 | LOC389906 | Homo sapiens zinc finger protein 839 pseudogene (LOC389906), non-coding RNA [NR_034031] |
| A_21_P0002579 | -2.7687716 |  |  |
| A_33_P3246153 | -2.7658956 |  | GB |
| A_33_P3335940 | -2.765448 | HN1L | Homo sapiens hematological and neurological expressed 1-like (HN1L), mRNA [NM_144570] |
| A_24_P84428 | -2.7653012 | CACYBP | Homo sapiens calcyclin binding protein (CACYBP), transcript variant 1, mRNA [NM_014412] |
| A_23_P204436 | -2.7634888 | GIT2 | Homo sapiens G protein-coupled receptor kinase interacting ArfGAP 2 (GIT2), transcript variant 4, mRNA [NM_139201] |
| A_33_P3245977 | -2.7614126 | SHROOM1 | Homo sapiens shroom family member 1 (SHROOM1), transcript variant 2, mRNA [NM_133456] |
| A_33_P3285038 | -2.7583995 | CERS5 | Homo sapiens ceramide synthase 5 (CERS5), transcript variant 2, mRNA [NM_001281731] |
| A_23_P57709 | -2.7547255 | PCOLCE2 | Homo sapiens procollagen C-endopeptidase enhancer 2 (PCOLCE2), mRNA [NM_013363] |
| A_23_P143535 | -2.7543871 | WDR4 | Homo sapiens WD repeat domain 4 (WDR4), transcript variant 2, mRNA [NM_033661] |
| A_21_P0010590 | -2.7469654 | LOC100288102 | PREDICTED: Homo sapiens uncharacterized LOC100288102 (LOC100288102), transcript variant X3, misc_RNA [XR_247074] |
| A_21_P0000270 | -2.7468696 | SNORA63 | Homo sapiens small nucleolar RNA, H/ACA box 63 (SNORA63), small nucleolar RNA [NR_002586] |
| A_21_P0000347 | -2.7398045 | SNORA76 | Homo sapiens small nucleolar RNA, H/ACA box 76 (SNORA76), small nucleolar RNA [NR_002995] |
| A_23_P377839 | -2.7391326 | LINC00161 | Homo sapiens long intergenic non-protein coding RNA 161 (LINC00161), transcript variant 1, long non-coding RNA [NR_026552] |
| A_23_P78557 | -2.736185 | FBXL12 | Homo sapiens F-box and leucine-rich repeat protein 12 (FBXL12), mRNA [NM_017703] |
| ERCC-00096_179 | -2.7361164 |  |  |
| A_33_P3334162 | -2.7353559 |  |  |
| A_33_P3280147 | -2.7339659 | FLJ45079 | Homo sapiens FLJ45079 protein (FLJ45079), long non-coding RNA [NR_028337] |
| A_21_P0004784 | -2.7265182 |  |  |
| A_32_P128310 | -2.7264853 | CDKAL1 | Homo sapiens CDK5 regulatory subunit associated protein 1-like 1 (CDKAL1), mRNA [NM_017774] |
| A_33_P3288079 | -2.725311 |  |  |
| A_24_P90881 | -2.7217307 | CES3 | Homo sapiens carboxylesterase 3 (CES3), transcript variant 1, mRNA [NM_024922] |
| A_33_P3224809 | -2.7214446 | IL17RA | Homo sapiens interleukin 17 receptor A (IL17RA), mRNA [NM_014339] |
| A_33_P3420367 | -2.7204716 | MIEF2 | Smith-Magenis syndrome chromosome region, candidate 7 [Source:HGNC Symbol;Acc:17920] [ENST00000395703] |
| A_32_P72341 | -2.7200081 | TRIM59 | Homo sapiens tripartite motif containing 59 (TRIM59), mRNA [NM_173084] |
| A_33_P3253902 | -2.719922 |  |  |
| A_33_P3382380 | -2.7168245 | UBQLN4 | Homo sapiens ubiquilin 4 (UBQLN4), mRNA [NM_020131] |
| A_23_P334751 | -2.7166295 | B3GALNT2 | Homo sapiens beta-1,3-N-acetylgalactosaminyltransferase 2 (B3GALNT2), transcript variant 1, mRNA [NM_152490] |
| A_33_P3250463 | -2.7139168 | PMS1 | Homo sapiens PMS1 postmeiotic segregation increased 1 (S. cerevisiae) (PMS1), transcript variant 3, mRNA [NM_001128144] |
| A_33_P3395196 | -2.7127206 |  | DA380124 BRTHA2 Homo sapiens cDNA clone BRTHA2013208 5', mRNA sequence [DA380124] |
| A_21_P0001300 | -2.7078478 |  |  |
| A_32_P217510 | -2.704106 | WDR75 | Homo sapiens WD repeat domain 75 (WDR75), mRNA [NM_032168] |
| (+)E1A_r60_a104 | -2.703054 |  | Unknown |
| A_33_P3266410 | -2.6990585 | MAZ | Homo sapiens MYC-associated zinc finger protein (purine-binding transcription factor) (MAZ), transcript variant 1, mRNA [NM_002383] |
| A_21_P0001288 | -2.6989384 |  |  |
| A_21_P0010834 | -2.6969657 | ZNF532 | Homo sapiens zinc finger protein 532 (ZNF532), mRNA [NM_018181] |
| A_24_P205213 | -2.689107 | ARSB | Homo sapiens arylsulfatase B (ARSB), transcript variant 2, mRNA [NM_198709] |
| A_21_P0012821 | -2.6872296 |  |  |
| A_23_P12053 | -2.684621 | SPEN | Homo sapiens spen family transcriptional repressor (SPEN), mRNA [NM_015001] |
| A_33_P3389673 | -2.6821954 | OR5A2 | Homo sapiens olfactory receptor, family 5, subfamily A, member 2 (OR5A2), mRNA [NM_001001954] |
| A_21_P0014716 | -2.6808095 | LRCH3 | leucine-rich repeats and calponin homology (CH) domain containing 3 [Source:HGNC Symbol;Acc:28637] [ENST00000536618] |
| A_21_P0010794 | -2.677926 | LOC100288102 | PREDICTED: Homo sapiens uncharacterized LOC101929827 (LOC101929827), transcript variant X2, misc_RNA [XR_254457] |
| A_21_P0004582 | -2.677828 |  |  |
| A_33_P3215232 | -2.6769392 |  |  |
| A_23_P110276 | -2.675973 | AFAP1-AS1 | Homo sapiens AFAP1 antisense RNA 1 (AFAP1-AS1), antisense RNA [NR_026892] |
| A_23_P104372 | -2.6655743 | DNAJC9 | Homo sapiens DnaJ (Hsp40) homolog, subfamily C, member 9 (DNAJC9), mRNA [NM_015190] |
| A_33_P3224426 | -2.6644688 | LOC729305 | Homo sapiens cDNA FLJ43050 fis, clone BRTHA3005046. [AK125040] |
| A_21_P0002348 | -2.6621616 |  |  |
| A_21_P0001251 | -2.6614244 | LOC101928895 | PREDICTED: Homo sapiens uncharacterized LOC101928895 (LOC101928895), misc_RNA [XR_241053] |
| A_21_P0011561 | -2.6610708 |  |  |
| A_21_P0008901 | -2.6610374 |  |  |
| A_33_P3276053 | -2.6589592 | LIMD1 | UI-HF-BN0-aed-f-06-0-UI.r1 NIH_MGC_50 Homo sapiens cDNA clone IMAGE:3064066 5', mRNA sequence [BU428864] |
| A_21_P0008582 | -2.655965 |  |  |
| A_21_P0011478 | -2.653179 |  | PREDICTED: Homo sapiens ribosome biogenesis protein BMS1 homolog (LOC101929959), transcript variant X1, mRNA [XM_005276349] |
| A_33_P3384885 | -2.649592 | ZNF511 | zinc finger protein 511 [Source:HGNC Symbol;Acc:28445] [ENST00000359035] |
| A_21_P0000236 | -2.649127 | SNORA64 | Homo sapiens small nucleolar RNA, H/ACA box 64 (SNORA64), small nucleolar RNA [NR_002326] |
| A_32_P55860 | -2.6488142 | SKA2 | Homo sapiens spindle and kinetochore associated complex subunit 2 (SKA2), transcript variant 1, mRNA [NM_182620] |
| A_21_P0000358 | -2.6482048 | SNORA71C | Homo sapiens small nucleolar RNA, H/ACA box 71C (SNORA71C), small nucleolar RNA [NR_003017] |
| A_33_P3269388 | -2.6480427 | MBOAT7 | Homo sapiens membrane bound O-acyltransferase domain containing 7 (MBOAT7), transcript variant 4, mRNA [NM_001146082] |
| A_33_P3279984 | -2.6451988 |  |  |
| A_33_P3298387 | -2.6440752 | PLK1 | Homo sapiens polo-like kinase 1 (PLK1), mRNA [NM_005030] |
| A_23_P85543 | -2.6422856 | RNF2 | Homo sapiens ring finger protein 2 (RNF2), mRNA [NM_007212] |
| A_33_P3406572 | -2.6386473 | APCDD1L-AS1 | APCDD1L antisense RNA 1 (head to head) [Source:HGNC Symbol;Acc:27152] [ENST00000447767] |
| A_33_P3383561 | -2.638577 | POLR2F | polymerase (RNA) II (DNA directed) polypeptide F [Source:HGNC Symbol;Acc:9193] [ENST00000443002] |
| A_33_P3729436 | -2.6329427 | SNAR-B1 | 602076178F1 NIH_MGC_62 Homo sapiens cDNA clone IMAGE:4243362 5', mRNA sequence [BF570972] |
| A_23_P107693 | -2.6323936 | ZNF586 | Homo sapiens zinc finger protein 586 (ZNF586), transcript variant 1, mRNA [NM_017652] |
| A_23_P96542 | -2.626 | VMA21 | Homo sapiens VMA21 vacuolar H+-ATPase homolog (S. cerevisiae) (VMA21), mRNA [NM_001017980] |
| A_21_P0004813 | -2.6241913 |  |  |
| A_33_P3284686 | -2.622246 | SSBP3 | Homo sapiens single stranded DNA binding protein 3 (SSBP3), transcript variant 1, mRNA [NM_145716] |
| A_23_P33433 | -2.6204433 | MAZ | Homo sapiens MYC-associated zinc finger protein (purine-binding transcription factor) (MAZ), transcript variant 2, mRNA [NM_001042539] |
| A_33_P3274701 | -2.6194925 |  | Homo sapiens cDNA FLJ14100 fis, clone MAMMA1000855. [AK024162] |
| A_23_P132388 | -2.6192524 | SCO2 | Homo sapiens SCO2 cytochrome c oxidase assembly protein (SCO2), transcript variant 1, mRNA [NM_005138] |
| A_24_P230176 | -2.6186934 | CCDC137 | Homo sapiens coiled-coil domain containing 137 (CCDC137), mRNA [NM_199287] |
| A_23_P168541 | -2.6153853 | C7orf26 | Homo sapiens chromosome 7 open reading frame 26 (C7orf26), mRNA [NM_024067] |
| A_21_P0006148 | -2.6143382 |  |  |
| A_24_P172993 | -2.6083057 | UBE2O | Homo sapiens ubiquitin-conjugating enzyme E2O (UBE2O), mRNA [NM_022066] |
| A_33_P3299386 | -2.6081023 | ZNF550 | Homo sapiens zinc finger protein 550 (ZNF550), transcript variant 3, mRNA [NM_001277092] |
| A_23_P39116 | -2.6055174 | LIG1 | Homo sapiens ligase I, DNA, ATP-dependent (LIG1), mRNA [NM_000234] |
| A_21_P0011890 | -2.6017797 |  |  |
| A_21_P0011430 | -2.5996585 | HERC2 | Homo sapiens HECT and RLD domain containing E3 ubiquitin protein ligase 2 (HERC2), mRNA [NM_004667] |
| ERCC-00043_129 | -2.5963058 |  | Unknown |
| A_24_P144773 | -2.5949867 | RNF145 | Homo sapiens ring finger protein 145 (RNF145), transcript variant 2, mRNA [NM_144726] |
| A_33_P3313597 | -2.5947154 | LOC100509861 | Protein LOC100509861 [Source:UniProtKB/TrEMBL;Acc:M0R1Y7] [ENST00000596861] |
| A_33_P3264771 | -2.5855713 | NOTO | Homo sapiens notochord homeobox (NOTO), mRNA [NM_001134462] |
| A_21_P0008716 | -2.5838263 |  |  |
| A_33_P3237948 | -2.5833206 | TCHHL1 | Homo sapiens trichohyalin-like 1 (TCHHL1), mRNA [NM_001008536] |
| A_21_P0011889 | -2.5815234 |  | PREDICTED: Homo sapiens uncharacterized LOC101927187 (LOC101927187), misc_RNA [XR_241443] |
| A_24_P273413 | -2.5789833 | EML4 | Homo sapiens echinoderm microtubule associated protein like 4 (EML4), transcript variant 1, mRNA [NM_019063] |
| A_33_P3375889 | -2.5789018 |  |  |
| A_23_P114314 | -2.578443 | CDX4 | Homo sapiens caudal type homeobox 4 (CDX4), mRNA [NM_005193] |
| A_33_P3375766 | -2.5777001 | KCNC2 | Homo sapiens potassium voltage-gated channel, Shaw-related subfamily, member 2 (KCNC2), transcript variant 3, mRNA [NM_153748] |
| A_21_P0000359 | -2.5769718 | SNORA71D | Homo sapiens small nucleolar RNA, H/ACA box 71D (SNORA71D), small nucleolar RNA [NR_003018] |
| A_21_P0000663 | -2.5747454 | LINC00622 | Homo sapiens long intergenic non-protein coding RNA 622 (LINC00622), long non-coding RNA [NR_036540] |
| A_23_P103628 | -2.5719156 | HEATR1 | Homo sapiens HEAT repeat containing 1 (HEATR1), mRNA [NM_018072] |
| A_23_P218646 | -2.564796 | TNFRSF6B | Homo sapiens tumor necrosis factor receptor superfamily, member 6b, decoy (TNFRSF6B), mRNA [NM_003823] |
| A_21_P0008988 | -2.5643268 |  |  |
| A_21_P0005979 | -2.56384 | SLC45A4 | Homo sapiens solute carrier family 45, member 4 (SLC45A4), transcript variant 1, mRNA [NM_001286646] |
| A_19_P00332515 | -2.5619369 | TSIX | Homo sapiens TSIX transcript, XIST antisense RNA (TSIX), antisense RNA [NR_003255] |
| A_19_P00804596 | -2.5587373 | C17orf51 | Homo sapiens chromosome 17 open reading frame 51 (C17orf51), mRNA [NM_001113434] |
| A_21_P0003375 | -2.5587306 |  |  |
| A_32_P809810 | -2.5561666 | LOC100130433 | Homo sapiens cDNA FLJ38936 fis, clone NT2NE2015275. [AK096255] |
| A_23_P17204 | -2.553961 | ANAPC1 | Homo sapiens anaphase promoting complex subunit 1 (ANAPC1), mRNA [NM_022662] |
| A_21_P0001865 | -2.553522 |  | DA767045 NTONG2 Homo sapiens cDNA clone NTONG2008452 5', mRNA sequence [DA767045] |
| A_21_P0001426 | -2.5507822 |  |  |
| A_33_P3345350 | -2.5501754 | DRGX | Homo sapiens dorsal root ganglia homeobox (DRGX), mRNA [NM_001276451] |
| ERCC-00154_60 | -2.5500412 |  |  |
| A_21_P0003708 | -2.548525 |  |  |
| A_23_P83835 | -2.5465808 | POMK | Homo sapiens protein-O-mannose kinase (POMK), transcript variant 1, mRNA [NM_032237] |
| A_23_P346421 | -2.5435421 | ZNF532 | Homo sapiens zinc finger protein 532 (ZNF532), mRNA [NM_018181] |
| A_24_P384755 | -2.5384316 | NOL10 | Homo sapiens nucleolar protein 10 (NOL10), transcript variant 1, mRNA [NM_024894] |
| A_23_P45864 | -2.5372863 | TNR | Homo sapiens tenascin R (TNR), mRNA [NM_003285] |
| A_21_P0000488 | -2.523385 | SNORD96B | Homo sapiens small nucleolar RNA, C/D box 96B (SNORD96B), small nucleolar RNA [NR_004379] |
| A_21_P0000323 | -2.5217144 | SNORA36A | Homo sapiens small nucleolar RNA, H/ACA box 36A (SNORA36A), small nucleolar RNA [NR_002969] |
| A_23_P345710 | -2.5212035 | XXYLT1 | Homo sapiens xyloside xylosyltransferase 1 (XXYLT1), mRNA [NM_152531] |
| A_33_P3278475 | -2.5208416 | FOXN2 | Homo sapiens forkhead box N2 (FOXN2), mRNA [NM_002158] |
| A_33_P3872301 | -2.519416 | SNAR-C3 | 602075775F1 NIH_MGC_62 Homo sapiens cDNA clone IMAGE:4242834 5', mRNA sequence [BF570763] |
| A_24_P212531 | -2.5128715 | B3GALNT2 | Homo sapiens beta-1,3-N-acetylgalactosaminyltransferase 2 (B3GALNT2), transcript variant 1, mRNA [NM_152490] |
| A_32_P113508 | -2.508227 | ATG16L1 | Homo sapiens autophagy related 16-like 1 (S. cerevisiae) (ATG16L1), transcript variant 1, mRNA [NM_030803] |
| A_23_P23411 | -2.5066988 | PRCC | Homo sapiens papillary renal cell carcinoma (translocation-associated) (PRCC), mRNA [NM_005973] |
| A_21_P0002235 | -2.49973 |  |  |
| A_33_P3257627 | -2.4982789 |  |  |
| A_21_P0001786 | -2.4944582 |  | long intergenic non-protein coding RNA 211 [Source:HGNC Symbol;Acc:37459] [ENST00000413792] |
| ERCC-00028_121 | -2.4887455 |  | Unknown |
| A_33_P3421203 | -2.4885528 | FAM86B3P | Homo sapiens family with sequence similarity 86, member B3, pseudogene (FAM86B3P), transcript variant 1, non-coding RNA [NR_024362] |
| A_21_P0010231 | -2.4816973 |  |  |
| A_33_P3727762 | -2.4815578 | SET | Homo sapiens SET nuclear oncogene (SET), transcript variant 2, mRNA [NM_003011] |
| A_33_P3229587 | -2.481287 |  |  |
| A_33_P3214199 | -2.4806142 | ZNF532 | Homo sapiens zinc finger protein 532 (ZNF532), mRNA [NM_018181] |
| A_21_P0011477 | -2.4800394 | BMS1P17 | Homo sapiens BMS1 pseudogene 17 (BMS1P17), non-coding RNA [NR_073460] |
| A_33_P3232828 | -2.479319 | SRSF3 | Homo sapiens serine/arginine-rich splicing factor 3 (SRSF3), transcript variant 1, mRNA [NM_003017] |
| A_33_P3383189 | -2.4788065 | SP9 | Homo sapiens Sp9 transcription factor (SP9), mRNA [NM_001145250] |
| A_24_P159181 | -2.478627 | PDCD7 | Homo sapiens programmed cell death 7 (PDCD7), mRNA [NM_005707] |
| A_23_P253586 | -2.4774723 | DOPEY2 | Homo sapiens dopey family member 2 (DOPEY2), mRNA [NM_005128] |
| A_33_P3211263 | -2.4766366 | GPATCH4 | Homo sapiens G patch domain containing 4 (GPATCH4), transcript variant 2, mRNA [NM_182679] |
| A_33_P3405399 | -2.471708 | ODF2L | Homo sapiens outer dense fiber of sperm tails 2-like (ODF2L), transcript variant 3, mRNA [NM_001184765] |
| A_19_P00801041 | -2.4711158 |  |  |
| A_23_P134729 | -2.4678524 | RBM12B-AS1 | Homo sapiens RBM12B antisense RNA 1 (RBM12B-AS1), long non-coding RNA [NR_027259] |
| A_23_P46309 | -2.4610083 | RCC1 | Homo sapiens regulator of chromosome condensation 1 (RCC1), transcript variant 4, mRNA [NM_001048199] |
| A_33_P3362249 | -2.4592726 | LOC100996380 | PREDICTED: Homo sapiens putative uncharacterized protein FLJ46348-like (LOC100996380), mRNA [XM_005250738] |
| A_33_P3256088 | -2.4591913 | LOC729218 | Homo sapiens uncharacterized LOC729218 (LOC729218), non-coding RNA [NR_103825] |
| A_33_P3407937 | -2.4582407 | PLCXD1 | Homo sapiens phosphatidylinositol-specific phospholipase C, X domain containing 1 (PLCXD1), transcript variant 1, mRNA [NM_018390] |
| A_21_P0012381 | -2.4580636 |  |  |
| A_23_P3355 | -2.456603 | POLG | Homo sapiens polymerase (DNA directed), gamma (POLG), transcript variant 1, mRNA [NM_002693] |
| A_23_P425104 | -2.4564142 | BRD4 | Homo sapiens bromodomain containing 4 (BRD4), transcript variant long, mRNA [NM_058243] |
| A_33_P3321120 | -2.4553866 |  |  |
| A_21_P0006469 | -2.451375 |  |  |
| A_33_P3229375 | -2.4503942 | PSMB11 | Homo sapiens proteasome (prosome, macropain) subunit, beta type, 11 (PSMB11), mRNA [NM_001099780] |
| A_24_P186346 | -2.4454896 | ANAPC1 | Homo sapiens anaphase promoting complex subunit 1 (ANAPC1), mRNA [NM_022662] |
| A_33_P3349693 | -2.4446785 | LCLAT1 | Homo sapiens lysocardiolipin acyltransferase 1 (LCLAT1), transcript variant 1, mRNA [NM_182551] |
| A_33_P3257538 | -2.4417741 | HYKK | Homo sapiens hydroxylysine kinase (HYKK), transcript variant 2, mRNA [NM_001083612] |
| A_24_P403734 | -2.4396973 | ZNF385A | Homo sapiens zinc finger protein 385A (ZNF385A), transcript variant 3, mRNA [NM_015481] |
| A_33_P3327971 | -2.4361825 |  |  |
| A_21_P0012973 | -2.4357421 |  | AGENCOURT_13890273 NIH_MGC_147 Homo sapiens cDNA clone IMAGE:30346469 5', mRNA sequence [CD110012] |
| A_21_P0006475 | -2.4344347 |  |  |
| A_23_P389588 | -2.4338176 | TCF7L2 | Homo sapiens transcription factor 7-like 2 (T-cell specific, HMG-box) (TCF7L2), transcript variant 2, mRNA [NM_030756] |
| A_33_P3409238 | -2.430369 | LOC401286 | PREDICTED: Homo sapiens uncharacterized LOC401286 (LOC401286), transcript variant X2, misc_RNA [XR_245638] |
| A_33_P3316313 | -2.4298224 | MTERFD2 | Homo sapiens MTERF domain containing 2 (MTERFD2), transcript variant 1, mRNA [NM_182501] |
| A_21_P0000324 | -2.4277694 | SNORA36A | Homo sapiens small nucleolar RNA, H/ACA box 36A (SNORA36A), small nucleolar RNA [NR_002969] |
| A_24_P781615 | -2.4236019 | AOC4P | Homo sapiens amine oxidase, copper containing 4, pseudogene (AOC4P), non-coding RNA [NR_002773] |
| A_33_P3330428 | -2.4221373 | FLJ44790 | PREDICTED: Homo sapiens uncharacterized FLJ44790 (FLJ44790), misc_RNA [XR_109635] |
| A_33_P3326349 | -2.4213433 | SMG5 | Homo sapiens SMG5 nonsense mediated mRNA decay factor (SMG5), mRNA [NM_015327] |
| A_21_P0012602 | -2.4210477 |  |  |
| A_24_P134235 | -2.4193919 | KHSRP | Homo sapiens KH-type splicing regulatory protein (KHSRP), mRNA [NM_003685] |
| A_33_P3370265 | -2.4160116 |  |  |
| A_33_P3421759 | -2.4159029 | SON | Homo sapiens SON DNA binding protein (SON), transcript variant b, mRNA [NM_032195] |
| A_33_P3287472 | -2.4152899 | PPP1R27 | Homo sapiens protein phosphatase 1, regulatory subunit 27 (PPP1R27), mRNA [NM_001007533] |
| A_24_P916718 | -2.4126225 | ZNF467 | Homo sapiens zinc finger protein 467 (ZNF467), mRNA [NM_207336] |
| A_33_P3299119 | -2.406169 | HNF1A | Homo sapiens HNF1 homeobox A (HNF1A), mRNA [NM_000545] |
| ERCC-00054_84 | -2.4029777 |  |  |
| ERCC-00039_95 | -2.399711 |  |  |
| A_23_P160742 | -2.398759 | GLMN | Homo sapiens glomulin, FKBP associated protein (GLMN), mRNA [NM_053274] |
| A_23_P143127 | -2.397117 | EML4 | Homo sapiens echinoderm microtubule associated protein like 4 (EML4), transcript variant 1, mRNA [NM_019063] |
| ETG10_234183 | -2.3888483 |  | Unknown |
| A_21_P0012798 | -2.3887105 | LINC00616 | long intergenic non-protein coding RNA 616 [Source:HGNC Symbol;Acc:44065] [ENST00000503208] |
| A_21_P0012366 | -2.3864832 | LOC101929530 | PREDICTED: Homo sapiens Fanconi anemia group D2 protein-like (LOC101929530), mRNA [XM_005275947] |
| A_23_P343261 | -2.3860564 | LINC00337 | Homo sapiens long intergenic non-protein coding RNA 337 (LINC00337), long non-coding RNA [NR_103534] |
| A_23_P217015 | -2.38304 | SET | Homo sapiens SET nuclear oncogene (SET), transcript variant 2, mRNA [NM_003011] |
| A_33_P3282261 | -2.3829806 |  | DKFZp434B0512_s1 434 (synonym: htes3) Homo sapiens cDNA clone DKFZp434B0512, mRNA sequence [AL039808] |
| A_23_P101774 | -2.3754356 | SLC5A5 | Homo sapiens solute carrier family 5 (sodium/iodide cotransporter), member 5 (SLC5A5), mRNA [NM_000453] |
| A_21_P0002534 | -2.3721187 |  |  |
| A_23_P45970 | -2.367021 | C1orf109 | Homo sapiens chromosome 1 open reading frame 109 (C1orf109), mRNA [NM_017850] |
| A_21_P0006287 | -2.3588192 |  |  |
| A_32_P26330 | -2.3563805 | USP10 | Homo sapiens ubiquitin specific peptidase 10 (USP10), transcript variant 2, mRNA [NM_005153] |
| A_33_P3286608 | -2.3538125 | PGBD2 | Homo sapiens piggyBac transposable element derived 2 (PGBD2), transcript variant 1, mRNA [NM_170725] |
| A_21_P0000605 | -2.3496392 | NSF | Homo sapiens N-ethylmaleimide-sensitive factor (NSF), transcript variant 1, mRNA [NM_006178] |
| A_23_P204736 | -2.3496048 | GPD1 | Homo sapiens glycerol-3-phosphate dehydrogenase 1 (soluble) (GPD1), transcript variant 1, mRNA [NM_005276] |
| A_33_P3282733 | -2.3486636 | LOC100131395 | PREDICTED: Homo sapiens uncharacterized LOC100131395 (LOC100131395), misc_RNA [XR_110093] |
| A_33_P3225600 | -2.3477788 | NFE2L2 | Homo sapiens nuclear factor, erythroid 2-like 2 (NFE2L2), transcript variant 1, mRNA [NM_006164] |
| ERCC-00012_90 | -2.3461099 |  | Unknown |
| A_32_P11450 | -2.3382578 | BMS1 | Homo sapiens BMS1 ribosome biogenesis factor (BMS1), mRNA [NM_014753] |
| A_21_P0005539 | -2.3381772 |  |  |
| A_24_P649747 | -2.3368938 | BMS1 | Homo sapiens BMS1 ribosome biogenesis factor (BMS1), mRNA [NM_014753] |
| A_33_P3213645 | -2.3364925 | ERN2 | Homo sapiens endoplasmic reticulum to nucleus signaling 2 (ERN2), mRNA [NM_033266] |
| A_21_P0000885 | -2.334454 | ZNF790-AS1 | Homo sapiens ZNF790 antisense RNA 1 (ZNF790-AS1), transcript variant 1, long non-coding RNA [NR_040027] |
| A_33_P3301025 | -2.331563 | CPOX | Homo sapiens coproporphyrinogen oxidase (CPOX), mRNA [NM_000097] |
| A_33_P3326827 | -2.3315554 |  |  |
| ERCC-00009_60 | -2.3292131 |  |  |
| A_21_P0005916 | -2.3263638 |  |  |
| A_23_P156445 | -2.323623 | DDX43 | Homo sapiens DEAD (Asp-Glu-Ala-Asp) box polypeptide 43 (DDX43), mRNA [NM_018665] |
| A_23_P213863 | -2.3216038 | FAM193B | Homo sapiens family with sequence similarity 193, member B (FAM193B), transcript variant 3, mRNA [NM_001190946] |
| A_21_P0009053 | -2.3193917 |  |  |
| A_21_P0010198 | -2.3177571 |  | BACH1 antisense RNA 1 [Source:HGNC Symbol;Acc:40008] [ENST00000449923] |
| A_23_P82823 | -2.31641 | PINX1 | Homo sapiens PIN2/TERF1 interacting, telomerase inhibitor 1 (PINX1), transcript variant 1, mRNA [NM_017884] |
| A_33_P3352134 | -2.3148825 | ATG16L1 | Homo sapiens autophagy related 16-like 1 (S. cerevisiae) (ATG16L1), transcript variant 1, mRNA [NM_030803] |
| A_21_P0013500 | -2.3145998 |  |  |
| A_23_P16139 | -2.3121347 | CHERP | Homo sapiens calcium homeostasis endoplasmic reticulum protein (CHERP), mRNA [NM_006387] |
| A_21_P0011431 | -2.310731 | HERC2 | Homo sapiens HECT and RLD domain containing E3 ubiquitin protein ligase 2 (HERC2), mRNA [NM_004667] |
| A_33_P3290487 | -2.308805 | ZNF668 | Homo sapiens zinc finger protein 668 (ZNF668), transcript variant 1, mRNA [NM_001172668] |
| A_33_P3317305 | -2.3086514 | AGAP1 | Homo sapiens ArfGAP with GTPase domain, ankyrin repeat and PH domain 1 (AGAP1), transcript variant 3, mRNA [NM_001244888] |
| A_23_P302681 | -2.3032262 | FIGNL1 | Homo sapiens fidgetin-like 1 (FIGNL1), transcript variant 1, mRNA [NM_001042762] |
| A_21_P0002445 | -2.3024802 |  |  |
| A_19_P00322416 | -2.3014483 | LINC00888 | Homo sapiens long intergenic non-protein coding RNA 888 (LINC00888), transcript variant 1, long non-coding RNA [NR_038301] |
| A_33_P3292699 | -2.3000646 |  | Homo sapiens LP8151 mRNA, complete cds. [AY203961] |
| A_23_P14193 | -2.298095 | RFC3 | Homo sapiens replication factor C (activator 1) 3, 38kDa (RFC3), transcript variant 1, mRNA [NM_002915] |
| A_33_P3382803 | -2.2980783 | LINC00643 | Homo sapiens long intergenic non-protein coding RNA 643 (LINC00643), long non-coding RNA [NR_015358] |
| A_24_P388252 | -2.294134 | PPP3R1 | Homo sapiens protein phosphatase 3, regulatory subunit B, alpha (PPP3R1), mRNA [NM_000945] |
| A_23_P57370 | -2.2940013 | CECR5 | Homo sapiens cat eye syndrome chromosome region, candidate 5 (CECR5), transcript variant 2, mRNA [NM_033070] |
| A_32_P191004 | -2.290847 | ATAD2B | Homo sapiens ATPase family, AAA domain containing 2B (ATAD2B), transcript variant 1, mRNA [NM_017552] |
| A_23_P334864 | -2.2898939 | FAM126B | Homo sapiens family with sequence similarity 126, member B (FAM126B), mRNA [NM_173822] |
| A_33_P3292417 | -2.2887025 | WDR5 | Homo sapiens WD repeat domain 5 (WDR5), transcript variant 1, mRNA [NM_017588] |
| A_24_P339664 | -2.2883973 | NCLN | Homo sapiens nicalin (NCLN), mRNA [NM_020170] |
| ERCC-00003_63 | -2.284394 |  |  |
| A_33_P3400477 | -2.28057 | STIL | Homo sapiens SCL/TAL1 interrupting locus (STIL), transcript variant 3, mRNA [NM_001282936] |
| A_21_P0007415 | -2.2792678 |  |  |
| A_33_P3330323 | -2.278466 | MGA | Homo sapiens MGA, MAX dimerization protein (MGA), transcript variant 2, mRNA [NM_001080541] |
| A_21_P0002697 | -2.2779927 | LINC00885 | Homo sapiens long intergenic non-protein coding RNA 885 (LINC00885), long non-coding RNA [NR_034088] |
| A_33_P3306828 | -2.2766182 | LOC100292952 | PREDICTED: Homo sapiens zinc finger protein 285-like (LOC100292952), mRNA [XM_005276120] |
| A_33_P3403102 | -2.2755005 | PRR18 | Homo sapiens proline rich 18 (PRR18), mRNA [NM_175922] |
| ERCC-00084_121 | -2.26903 |  |  |
| A_33_P3236272 | -2.2642817 | BANP | Homo sapiens BTG3 associated nuclear protein (BANP), transcript variant 3, mRNA [NM_001173539] |
| A_32_P226786 | -2.2618678 | FAM126B | Homo sapiens family with sequence similarity 126, member B (FAM126B), mRNA [NM_173822] |
| A_33_P3366124 | -2.2618542 |  |  |
| A_33_P3279109 | -2.2593706 | ZNF75A | zinc finger protein 75a [Source:HGNC Symbol;Acc:13146] [ENST00000575234] |
| A_24_P38895 | -2.255517 | H2AFX | Homo sapiens H2A histone family, member X (H2AFX), mRNA [NM_002105] |
| A_24_P933011 | -2.2538793 | TMEM200C | Homo sapiens transmembrane protein 200C (TMEM200C), mRNA [NM_001080209] |
| A_21_P0014276 | -2.2412825 |  |  |
| A_33_P3261308 | -2.2407787 |  |  |
| A_23_P319565 | -2.2394316 | PGBD3 | Homo sapiens piggyBac transposable element derived 3 (PGBD3), mRNA [NM_170753] |
| ERCC-00117_139 | -2.2384138 |  |  |
| A_23_P25433 | -2.2364557 | C12orf4 | Homo sapiens chromosome 12 open reading frame 4 (C12orf4), mRNA [NM_020374] |
| A_33_P3296198 | -2.2354305 | C5orf63 | Homo sapiens chromosome 5 open reading frame 63 (C5orf63), transcript variant 1, mRNA [NM_001164479] |
| A_21_P0008749 | -2.2260268 |  |  |
| A_33_P3376026 | -2.2245083 | LOC100128770 | Homo sapiens uncharacterized LOC100128770 (LOC100128770), long non-coding RNA [NR_047572] |
| A_19_P00316404 | -2.2232962 | C17orf51 | Homo sapiens chromosome 17 open reading frame 51 (C17orf51), mRNA [NM_001113434] |
| A_21_P0013956 | -2.2231567 | PPFIA4 | protein tyrosine phosphatase, receptor type, f polypeptide (PTPRF), interacting protein (liprin), alpha 4 [Source:HGNC Symbol;Acc:9248] [ENST00000601609] |
| A_33_P3316310 | -2.2229633 | MTERFD2 | Homo sapiens MTERF domain containing 2 (MTERFD2), transcript variant 1, mRNA [NM_182501] |
| A_21_P0004306 | -2.2208164 |  |  |
| A_33_P3240996 | -2.2202692 | FAM201B | PREDICTED: Homo sapiens family with sequence similarity 201, member B (FAM201B), misc_RNA [XR_158980] |
| A_23_P57413 | -2.2196112 | PPM1F | Homo sapiens protein phosphatase, Mg2+/Mn2+ dependent, 1F (PPM1F), mRNA [NM_014634] |
| A_23_P208143 | -2.2164671 | ZNF397 | Homo sapiens zinc finger protein 397 (ZNF397), transcript variant 1, mRNA [NM_001135178] |
| A_23_P68007 | -2.2163463 | ATP1B3 | Homo sapiens ATPase, Na+/K+ transporting, beta 3 polypeptide (ATP1B3), mRNA [NM_001679] |
| A_21_P0006883 | -2.2140818 |  |  |
| A_21_P0013456 | -2.2136528 |  |  |
| A_33_P3303385 | -2.2097712 | NCAPD2 | Homo sapiens non-SMC condensin I complex, subunit D2 (NCAPD2), mRNA [NM_014865] |
| A_33_P3401267 | -2.2068524 | FEZ2 | Homo sapiens fasciculation and elongation protein zeta 2 (zygin II) (FEZ2), transcript variant 2, mRNA [NM_001042548] |
| A_21_P0004064 | -2.2000096 |  |  |
| A_24_P258051 | -2.1978226 | MASTL | Homo sapiens microtubule associated serine/threonine kinase-like (MASTL), transcript variant 2, mRNA [NM_032844] |
| A_24_P211206 | -2.1974611 | TPM3 | Homo sapiens tropomyosin 3 (TPM3), transcript variant 3, mRNA [NM_001043352] |
| A_33_P3421338 | -2.1937828 | KIF18B | Homo sapiens kinesin family member 18B (KIF18B), transcript variant 1, mRNA [NM_001265577] |
| A_19_P00318768 | -2.192819 | LINC00630 | Homo sapiens long intergenic non-protein coding RNA 630 (LINC00630), long non-coding RNA [NR_038988] |
| A_33_P3222659 | -2.1916845 | LOC101929847 | PREDICTED: Homo sapiens golgin subfamily A member 2-like protein 3-like (LOC101929847), transcript variant X1, mRNA [XM_005276475] |
| A_21_P0000329 | -2.191091 | SNORA42 | Homo sapiens small nucleolar RNA, H/ACA box 42 (SNORA42), small nucleolar RNA [NR_002974] |
| A_21_P0012605 | -2.1901567 | CEP170 | Homo sapiens centrosomal protein 170kDa (CEP170), transcript variant alpha, mRNA [NM_014812] |
| A_23_P212715 | -2.1894875 | CBLB | Homo sapiens Cbl proto-oncogene B, E3 ubiquitin protein ligase (CBLB), mRNA [NM_170662] |
| A_33_P3214939 | -2.189382 | GOLGA6L5P | Homo sapiens golgin A6 family-like 5, pseudogene (GOLGA6L5P), non-coding RNA [NR_003246] |
| A_33_P3356004 | -2.185528 | UCKL1-AS1 | Homo sapiens UCKL1 antisense RNA 1 (UCKL1-AS1), antisense RNA [NR_027287] |
| A_21_P0012401 | -2.1855104 |  |  |
| A_23_P320658 | -2.1835494 | BUB3 | Homo sapiens BUB3 mitotic checkpoint protein (BUB3), transcript variant 1, mRNA [NM_004725] |
| A_21_P0014205 | -2.1807072 | WBSCR22 | Williams Beuren syndrome chromosome region 22 [Source:HGNC Symbol;Acc:16405] [ENST00000423166] |
| A_33_P3223825 | -2.1757789 | SRRM3 | Homo sapiens serine/arginine repetitive matrix 3 (SRRM3), mRNA [NM_001110199] |
| A_33_P3365747 | -2.172473 | LINC00265 | Homo sapiens long intergenic non-protein coding RNA 265 (LINC00265), long non-coding RNA [NR_026999] |
| A_33_P3329592 | -2.1720455 | BLOC1S3 | Homo sapiens biogenesis of lysosomal organelles complex-1, subunit 3 (BLOC1S3), mRNA [NM_212550] |
| A_33_P3243683 | -2.1715496 | DIAPH1 | Homo sapiens diaphanous-related formin 1 (DIAPH1), transcript variant 1, mRNA [NM_005219] |
| ERCC-00098_150 | -2.1656528 |  |  |
| A_33_P3364607 | -2.1611972 | LOC100130051 | Homo sapiens cDNA FLJ45363 fis, clone BRHIP3015854. [AK127296] |
| A_33_P3293529 | -2.1574557 |  |  |
| A_33_P3400292 | -2.145389 |  |  |
| A_33_P3355856 | -2.144357 |  | olfactory receptor, family 51, subfamily J, member 1 (gene/pseudogene) [Source:HGNC Symbol;Acc:14856] [ENST00000332043] |
| A_21_P0000161 | -2.1440666 | USB1 | Homo sapiens U6 snRNA biogenesis 1 (USB1), transcript variant 3, mRNA [NM_001204911] |
| A_33_P3405360 | -2.1435285 | PDCL | Homo sapiens phosducin-like (PDCL), mRNA [NM_005388] |
| A_23_P63847 | -2.1424124 | SUPV3L1 | Homo sapiens suppressor of var1, 3-like 1 (S. cerevisiae) (SUPV3L1), mRNA [NM_003171] |
| A_32_P1445 | -2.1409729 | PTPN2 | Homo sapiens protein tyrosine phosphatase, non-receptor type 2 (PTPN2), transcript variant 3, mRNA [NM_080423] |
| A_23_P86386 | -2.1403391 | ZNF669 | Homo sapiens zinc finger protein 669 (ZNF669), transcript variant 1, mRNA [NM_024804] |
| A_33_P3380587 | -2.138639 | AGO1 | Homo sapiens argonaute RISC catalytic component 1 (AGO1), mRNA [NM_012199] |
| A_21_P0000135 | -2.1344435 | ANKHD1 | Homo sapiens ankyrin repeat and KH domain containing 1 (ANKHD1), transcript variant 3, mRNA [NM_024668] |
| ERCC-00034_158 | -2.1279128 |  |  |
| A_33_P3399693 | -2.1278353 | LOC100128374 | Protein LOC100128374 [Source:UniProtKB/TrEMBL;Acc:E9PF64] [ENST00000480694] |
| A_33_P3220095 | -2.1234467 | ZNF341 | Homo sapiens zinc finger protein 341 (ZNF341), transcript variant 1, mRNA [NM_001282933] |
| A_23_P251893 | -2.1203344 | BRAT1 | Homo sapiens BRCA1-associated ATM activator 1 (BRAT1), mRNA [NM_152743] |
| A_21_P0011968 | -2.1185367 |  |  |
| A_23_P88873 | -2.1178908 | GAN | Homo sapiens gigaxonin (GAN), mRNA [NM_022041] |
| A_33_P3272080 | -2.116824 | QRFP | oe35d02.y1 Human keratoconus cornea, unamplified, (od [CV575364] |
| A_24_P223163 | -2.1137002 | NAF1 | Homo sapiens nuclear assembly factor 1 ribonucleoprotein (NAF1), transcript variant 1, mRNA [NM_138386] |
| A_19_P00316493 | -2.109828 |  | DA414427 BRTHA3 Homo sapiens cDNA clone BRTHA3022791 5', mRNA sequence [DA414427] |
| A_23_P1641 | -2.1093502 | RCE1 | Homo sapiens Ras converting CAAX endopeptidase 1 (RCE1), transcript variant 1, mRNA [NM_005133] |
| A_32_P507710 | -2.109191 | PI4KAP2 | Homo sapiens phosphatidylinositol 4-kinase, catalytic, alpha pseudogene 2 (PI4KAP2), non-coding RNA [NR_003700] |
| A_23_P103942 | -2.1067889 | DNAJC11 | Homo sapiens DnaJ (Hsp40) homolog, subfamily C, member 11 (DNAJC11), mRNA [NM_018198] |
| A_21_P0013558 | -2.1057036 |  |  |
| A_33_P3416583 | -2.1034734 | ARAP1 | Homo sapiens ArfGAP with RhoGAP domain, ankyrin repeat and PH domain 1 (ARAP1), transcript variant 4, mRNA [NM_001135190] |
| A_33_P3245248 | -2.103253 | TERC | Homo sapiens telomerase RNA component (TERC), telomerase RNA [NR_001566] |
| A_23_P344037 | -2.1000423 | CHFR | Homo sapiens checkpoint with forkhead and ring finger domains, E3 ubiquitin protein ligase (CHFR), transcript variant 4, mRNA [NM_018223] |
| A_23_P115046 | -2.0997188 | EIF2B3 | Homo sapiens eukaryotic translation initiation factor 2B, subunit 3 gamma, 58kDa (EIF2B3), transcript variant 1, mRNA [NM_020365] |
| A_23_P215675 | -2.0969193 | COA1 | Homo sapiens cytochrome c oxidase assembly factor 1 homolog (S. cerevisiae) (COA1), transcript variant 1, mRNA [NM_018224] |
| A_23_P22915 | -2.0902915 | SLC30A7 | Homo sapiens solute carrier family 30 (zinc transporter), member 7 (SLC30A7), transcript variant 1, mRNA [NM_133496] |
| A_23_P8906 | -2.0893176 | LRP12 | Homo sapiens low density lipoprotein receptor-related protein 12 (LRP12), transcript variant 1, mRNA [NM_013437] |
| A_32_P109704 | -2.0842144 | SFSWAP | Homo sapiens splicing factor, suppressor of white-apricot homolog (Drosophila) (SFSWAP), transcript variant 2, mRNA [NM_004592] |
| A_23_P157416 | -2.0841045 | ZNF394 | Homo sapiens zinc finger protein 394 (ZNF394), mRNA [NM_032164] |
| A_21_P0000511 | -2.0837545 | SNAR-H | Homo sapiens small ILF3/NF90-associated RNA H (SNAR-H), small nuclear RNA [NR_024342] |
| A_33_P3387931 | -2.079035 | CENPP | Homo sapiens centromere protein P (CENPP), transcript variant 1, mRNA [NM_001012267] |
| A_33_P3294946 | -2.078096 | 1060P11.3 | Homo sapiens mRNA for killer-cell Ig-like receptor (KIR3DP1 gene), allele KIR3DP1*004. [AJ630586] |
| A_33_P3299130 | -2.076535 |  |  |
| A_33_P3362668 | -2.0600393 | LOC100132352 | Homo sapiens FSHD region gene 1 pseudogene (LOC100132352), non-coding RNA [NR_034006] |
| A_24_P315444 | -2.0566134 |  |  |
| A_21_P0014729 | -2.0548413 | POLR2J4 | Homo sapiens polymerase (RNA) II (DNA directed) polypeptide J4, pseudogene, mRNA (cDNA clone IMAGE:4867986). [BC017341] |
| A_23_P16573 | -2.0529466 | DDX49 | Homo sapiens DEAD (Asp-Glu-Ala-Asp) box polypeptide 49 (DDX49), transcript variant 1, mRNA [NM_019070] |
| A_33_P3415560 | -2.050351 | ZFP82 | Homo sapiens ZFP82 zinc finger protein (ZFP82), mRNA [NM_133466] |
| A_23_P424316 | -2.044528 | TCF20 | Homo sapiens transcription factor 20 (AR1) (TCF20), transcript variant 1, mRNA [NM_005650] |
| A_23_P16157 | -2.0437799 | KHSRP | Homo sapiens KH-type splicing regulatory protein (KHSRP), mRNA [NM_003685] |
| A_33_P3319486 | -2.043233 |  |  |
| A_23_P165657 | -2.0428507 | SLC20A1 | Homo sapiens solute carrier family 20 (phosphate transporter), member 1 (SLC20A1), mRNA [NM_005415] |
| A_33_P3365810 | -2.0421731 | MRPL12 | Homo sapiens mitochondrial ribosomal protein L12 (MRPL12), mRNA [NM_002949] |
| A_33_P3209346 | -2.0350997 | IARS | Homo sapiens isoleucyl-tRNA synthetase (IARS), transcript variant 2, mRNA [NM_013417] |
| A_23_P45851 | -2.033199 | HIAT1 | Homo sapiens hippocampus abundant transcript 1 (HIAT1), mRNA [NM_033055] |
| A_33_P3235521 | -2.0324872 |  |  |
| ERCC-00071_128 | -2.0322826 |  |  |
| A_21_P0000508 | -2.0316572 | SNAR-D | Homo sapiens small ILF3/NF90-associated RNA D (SNAR-D), small nuclear RNA [NR_024243] |
| ERCC-00109_120 | -2.0305839 |  |  |
| A_24_P269895 | -2.0190554 | HNRNPA3 | Homo sapiens heterogeneous nuclear ribonucleoprotein A3 (HNRNPA3), mRNA [NM_194247] |
| A_33_P3308101 | -2.0189986 |  | PREDICTED: Homo sapiens helicase SRCAP-like (LOC101929190), misc_RNA [XR_242363] |
| A_33_P3256828 | -2.0131218 | LOC100127910 | Homo sapiens cDNA FLJ27201 fis, clone SYN03133. [AK130711] |
| ERCC-00079_170 | -2.009653 |  |  |
| A_32_P831181 | -2.0093145 | BRI3BP | Homo sapiens BRI3 binding protein (BRI3BP), mRNA [NM_080626] |
| A_32_P109572 | -2.008699 | HNRNPL | Homo sapiens heterogeneous nuclear ribonucleoprotein L (HNRNPL), transcript variant 1, mRNA [NM_001533] |
| A_23_P101461 | -2.0069275 | CCDC130 | Homo sapiens coiled-coil domain containing 130 (CCDC130), mRNA [NM_030818] |
| A_23_P79231 | -2.0026994 | CREB1 | Homo sapiens cAMP responsive element binding protein 1 (CREB1), transcript variant B, mRNA [NM_134442] |
| A_21_P0013023 | -2.0023146 | RAB44 | Homo sapiens RAB44, member RAS oncogene family (RAB44), mRNA [NM_001257357] |
